# Posttranscriptional control of hepatic CEACAM1 3**′**UTR by human antigen R (HuR) mitigates sterile liver inflammation

**DOI:** 10.1172/jci.insight.194227

**Published:** 2025-09-23

**Authors:** Brian Cheng, Tristan D. Tibbe, Siyuan Yao, Megan Wei, Zeriel Y. Wong, Taylor Torgerson, Richard Chiu, Aanchal S. Kasargod, Kojiro Nakamura, Monica Cappelletti, Myung Sim, Douglas G. Farmer, Fady Kaldas, Jerzy W. Kupiec-Weglinski, Kenneth J. Dery

**Affiliations:** 1 The Dumont-UCLA Transplantation Center, Department of Surgery, Division of Liver and Pancreas Transplantation, and; 2 Department of Medicine Statistics Core, David Geffen School of Medicine at UCLA, Los Angeles, California, USA.; 3 Department of Pathology and Laboratory Medicine, UCLA Immunogenetics Center, Los Angeles, California, USA.

**Keywords:** Hepatology, Immunology, Inflammation, Hypoxia, Innate immunity, Organ transplantation

## Abstract

Hepatic ischemia-reperfusion injury (IRI) disrupts cellular signaling pathways and contributes to early allograft dysfunction (EAD) in orthotopic liver transplantation (OLT). In this study, we found that the hepatic RNA binding protein Human Antigen R (HuR) regulated the 3′ untranslated region (UTR) of Carcinoembryonic Antigen-Related Cell Adhesion Molecule 1 (*Ceacam1*) following ischemic stress. Hepatocyte-specific preinjury HuR-null mice exhibited elevated LDH-5 isoenzyme activity and reduced Ceacam1-S expression, reflecting tissue-specific injury. In situ hybridization demonstrated that the stability of *Ceacam1* mRNA depended on HuR. Luciferase assays identified *Ceacam1* 3′UTR *cis*-elements responsive to high oxygen tension. HuR-targeting short-activating RNAs (saRNAs) preferentially induced the alternative splicing of *Ceacam1-S*. Antisense oligos directed to the Ceacam1 3′UTR protected WT mice against acute liver injury. In the clinical arm, increased HuR and CEACAM1 expression were associated with reduced proinflammatory phenotype and a lower incidence of EAD in patients with OLT (*n* = 164). Human discarded livers with elevated *ELAVL1*/*CEACAM1* levels correlated with improved tissue homeostasis. These findings suggest that HuR regulation of Ceacam1 represents a key determinant of donor tissue quality and offers a potential target for future therapeutic strategies in OLT recipients.

## Graphical abstract

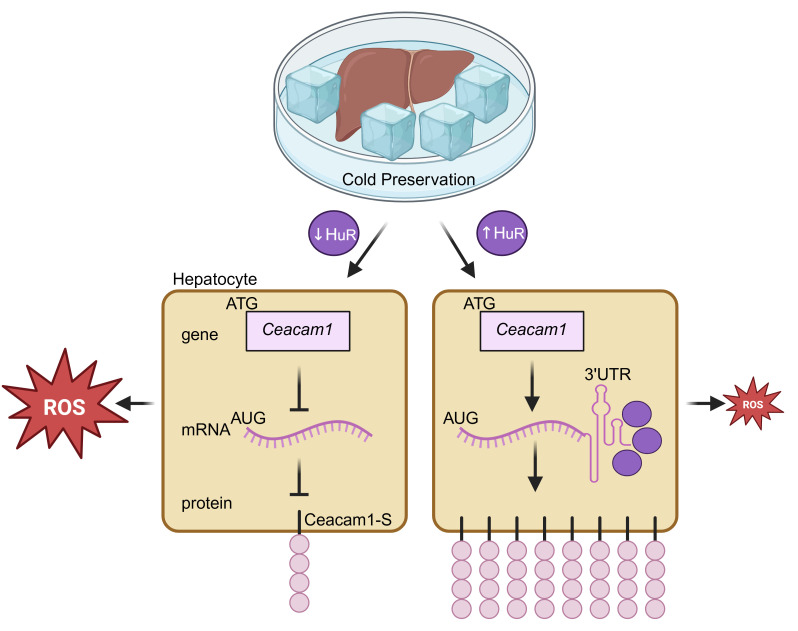

## Introduction

The shortage of donor organs continues to represent a challenging problem in clinical orthotopic liver transplantation (OLT) (1). Efforts to increase organ donation have included expanding the criteria into higher-risk categories, such as older donors with more comorbidities and donation after cardiac death (DCD). These marginal organs, however, are at increased risk of ischemia-reperfusion injury (IRI), an inevitable event in organ procurement from cadaver sources (2). IRI is an innate immune-driven sterile inflammation triggered by the temporary loss and subsequent restoration of blood flow, leading to hepatocellular damage and impaired graft function (3). Restoring the function of human livers that are discarded from transplantation due to marginal graft quality may be a viable strategy to expand the donor pool and reduce wait list mortality.

Our previous studies have determined that murine liver-specific Ceacam1 safeguards against cell death–triggered ASK1/p38 MAPK in hepatic IRI (4). This cytoprotection reflects the antiinflammatory regulatory functions of transmembrane glycoprotein CEACAM1 (encoded by the mu/hu *Ceacam1*/*CEACAM1* gene; *CD66a*) signaling in stressed hepatocytes (5) as well as activated neutrophils (6) and T cells (7). Extensive alternative splicing of CEACAM1 generates 2 divergent cytoplasmic domains (Ceacam1-S [short] or Ceacam1-L [long]) that confer specific intracellular signaling in epithelial, vascular, immune, and cancer cells (8–11).

Our recent data show that hepatocytes expressing Ceacam1-S under hypoxia/reoxygenation (H/R) and cold stress conditions are protected from cell death, highlighting the protective role of alternative splicing induction in stressed hepatocytes (5). In another study, we observed increased hepatocellular damage following Ceacam1-deficient liver transplantation into Ceacam1-proficient recipients. This suggested that the absence of CEACAM1 in donor hepatocytes may exacerbate liver injury during reperfusion, likely by failing to regulate the host immune response (7). Specifically, Ceacam1-deficient donor livers may lack the ability to interact in *trans* with CEACAM1 and T cell immunoglobulin and mucin domain-containing protein 3 (Tim-3) expressed on recipient T cells — ligands known to modulate favorable immune responses to inflammation and tissue injury (12). So far, whether hepatic Ceacam1 participates in heterophilic interactions with infiltrating immune cells during IRI remains unclear.

Developing small antisense oligonucleotides (ASOs) that promote Ceacam1 expression may provide a targeted strategy to assess Ceacam1’s functional role in immune crosstalk. ASOs are short, synthetic nucleic acid sequences that bind RNA and modulate gene expression (13). In our recent study, we used phosphorodiamidate morpholino oligomers (MO) to promote alternative splicing to enhance the expression of Ceacam1-S (5). MOs are synthetic ASOs that sterically hinder access to the target RNA. Alternatively, identifying molecular regulators of Ceacam1 during IRI may offer a complementary strategy to boost its protective function in hepatocytes. To that end, we recently showed that hepatic HuR positively regulates antioxidant heme oxygenase-1 (HO-1) through its 3′UTR to silence neutrophil proinflammatory cytokine/chemokine phenotype during the resolution of liver IRI (14). Human Antigen R (HuR; gene *ELAVL1*) is an RNA-binding stabilizer of adenylate-uridylate–rich (AU-rich) stretches typically found in the 3′UTR of mRNAs (15). The potential interplay of HuR regulation on antiinflammatory Ceacam1 function has not been determined and warrants further investigation.

In the present study, we identified Ceacam1 as a bona fide substrate of HuR. Mechanistic studies using HuR-targeting short-activating RNAs (saRNAs) and Ceacam1-3′UTR–targeted MOs protected hepatocytes from high-oxygen stress. In a clinical cohort of 164 patients with OLT, enhanced hepatic biopsy *CEACAM1/ELAVL1* expression pattern correlated with a decreased proinflammatory phenotype. HUR regulation of CEACAM1 was associated with improved early graft performance, as reflected by its influence on early allograft dysfunction (EAD) incidence. Finally, *CEACAM1/ELAVL1* expression was also associated with reduced proinflammatory gene signatures in discarded human livers deemed unsuitable for clinical OLT. Together, these findings identify HuR as a posttranscriptional regulator of Ceacam1, highlighting a previously unknown relationship that may be targetable to modulate hepatic responses to IRI in OLT recipients.

## Results

### Hepatic HuR-null mutation augments cytotoxic stress, leading to the loss of Ceacam1-S.

**Figure 1:**
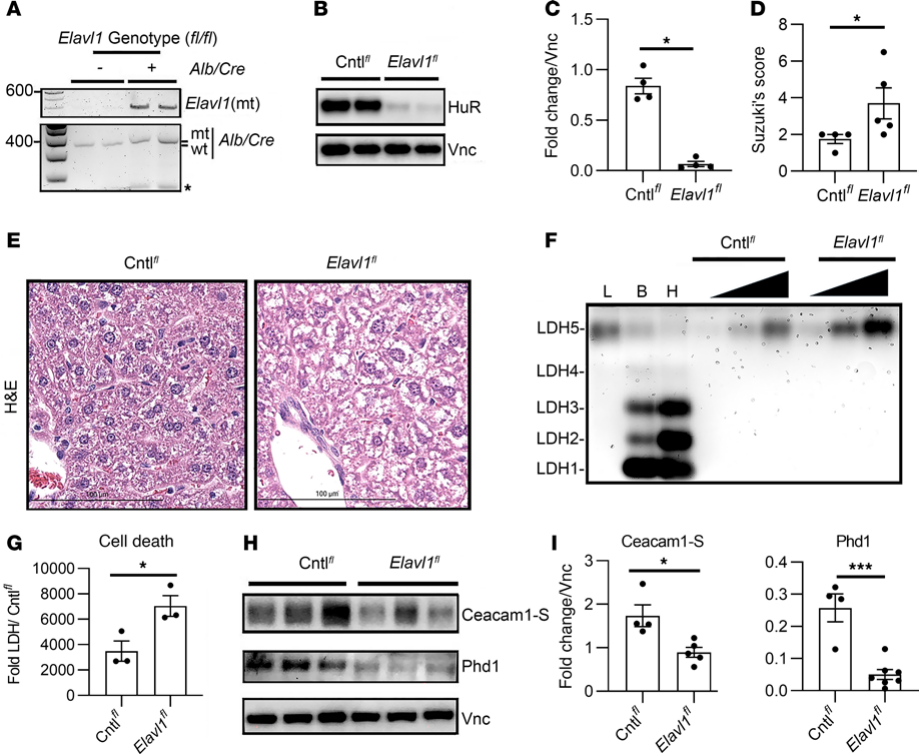
Hepatic HuR-null mutation augments cytotoxic stress, leading to loss of Ceacam1-S. (**A**) Representative RT-PCR of genomic DNA from HuR-KO mice (genotype floxed [*^fl/fl^*] *Elavl1,* Alb/Cre^+^). Littermates that contained only the floxed *Elavl1* mutation served as controls (Cntl*^fl^*). Mutant (mt) and WT notations refer to the zygosity of the *Elavl1* and *AlbCre* loci. Asterisk depicts nonspecific amplicon products. (**B**) Representative immunoblots of floxed control or *Elavl1^fl^* naive livers evaluated for HuR or Vnc expression. (**C**) Quantitation of HuR expression, relative Vnc (*n* = 4/group). (**D**) Suzuki’s histological grading (*n* = 4–5/group). (**E**) Representative H&E staining of floxed control or *Elavl1^fl^* naive livers. Scale bar: 100 μm. (**F**) LDH agarose isoenzyme assay of Cntl*^fl^* or *Elavl1^fl^* naive livers (*n* = 3/group). Mouse liver (L), brain (B), and heart (H) were used as migration controls. Black triangles denotes increasing volume of protein lysates from Cntl*^fl^* or *Elavl1^fl^* livers loaded (5, 10, or 20 μL for each). (**G**) Quantitation of the average from 3 biological replicates, across all volumes loaded, for the LDH-5 isoform relative to Cntl*^fl^* livers. (**H** and **I**) Representative Western blot and quantitation of Ceacam1-S, PHD1, and Vnc expression (*n* = 4–7/group). Vnc served as a loading control. Two-tailed Mann-Whitney *U* analyses were performed for **C** and **D**, and unpaired parametric 2-tailed Student’s *t* tests were performed for **G** and **I**. Data expressed are from at least 3 independent experiments (data are shown as mean ± SEM). *P < 0.05, ***P < 0.001.

We aimed to determine how graft-specific HuR signaling may affect the severity of liver injury and the cellular adaptation of Ceacam1 during temperature-dependent oxidative stress. Owing to the lethality of homozygous *Elavl1* embryos, we used a conditionally defective mouse HuR/*Elavl1* allele containing target sites for the Cre/LoxP recombination system (16, 17). Pups that coexpressed the *Elavl1* floxed (*Elavl1^fl^)* allele and were hemizygous or homozygous for the *AlbCre* (Albumin-Cre) recombinase allele (Figure 1A) showed low hepatic HuR protein levels in the naive state (Figure 1, B and C; *P* = 0.03, *Elavl1^fl^* versus floxed controls [Cntl*^fl^*] lacking *AlbCre*). These animals also showed enhanced hepatic necrosis as measured by Suzuki’s histological scoring of liver injury (*P* = 0.0397) (Figure 1, D and E). Lactate dehydrogenase (LDH) isoenzyme analysis was performed to separate isoforms based on their differential migration. Compared with floxed control livers, *Elavl1^fl^* showed a marked increase in LDH-5, the isoform associated with anaerobic glycolysis in mouse liver tissues (Figure 1, F and G; *P* = 0.0353).

Notably, hepatic HuR-deficient livers showed a marked decrease in Ceacam1-S (Figure 1, H and I; *P* = 0.013, *Elavl1^fl^* versus Cntl*^fl^*) and Prolyl Hydroxylase Domain-1 (Phd1) levels (Figure 1, H and I; *P* = 0.0004, *Elavl1^fl^* versus Cntl*^fl^*) in preinjury mice. We tested the Ceacam1-S isoform because our recent study showed that induction of its alternative splicing led to the protection of hepatocytes from hypoxic stress (5). Phd1 levels were tested because our recent study also implicated the oxygen-sensing HIF-1α transcription factor in the alternative splicing of *Ceacam1* (5). Phd proteins stabilize hypoxia-inducible factor (HIF) proteins under basal oxygen conditions (18). Collectively, these data implicate hepatic HuR in the positive control of hepatic *Ceacam1* gene expression profile through oxygen-sensing regulatory pathways that involve HIF-1α signaling.

### Hepatic Ceacam1 mRNA depends on HuR during temperature-dependent cellular stress.

**Figure 2:**
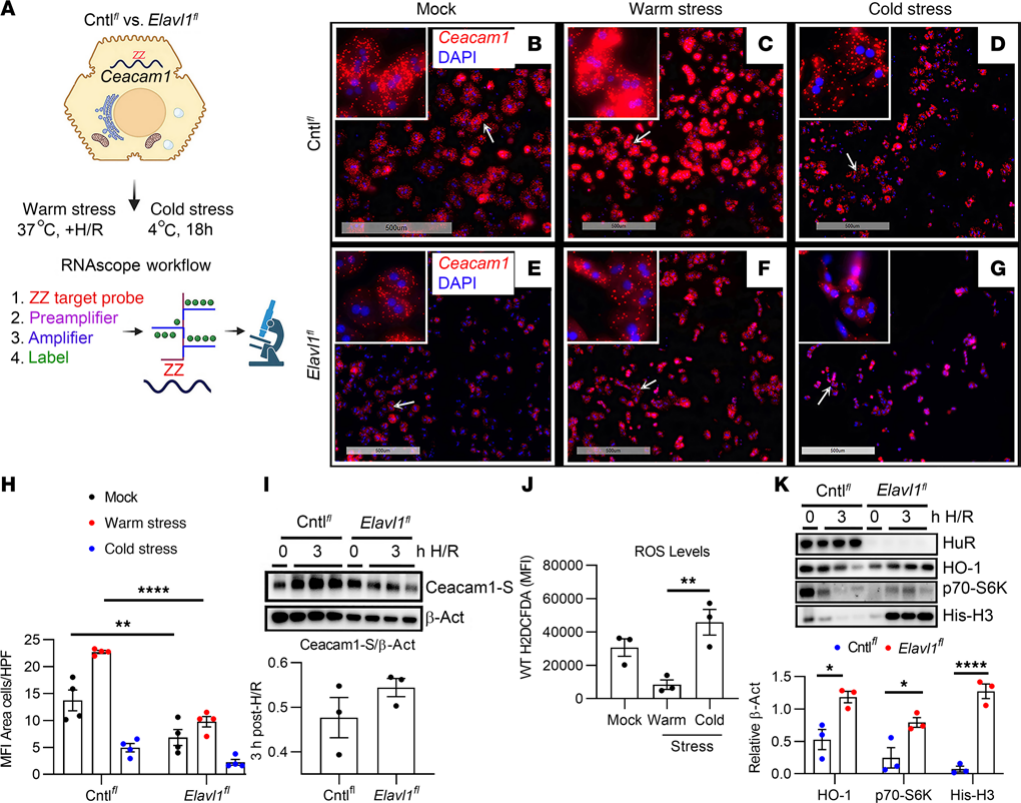
Hepatic *Ceacam1* mRNA depends on HuR signaling during temperature-dependent cellular stress. (**A**) Schematic RNAscope in situ hybridization of *Ceacam1* mRNAs at the single-cell resolution. Floxed control and *Elavl1^fl^* mouse hepatocytes were treated with warm (H/R was 3-hour hypoxia, 18-hour rexoygenation) versus cold stress. The workflow shows the probe platform built to amplify the signal. (**B**–**G**) Representative distribution of hepatocyte-derived *Ceacam1* (red) mRNA^+^ cells; nuclear (DAPI) staining (blue). Scale bar: 500 μm. Arrows show the selected inset image where the original magnification is scaled upward 150× for visualization. (**H**) Quantitation of mean fluorescent intensity (MFI) area of cells/ *n* = 4 high-power field (HPF). (**I**) Western blots/quantification of Ceacam1-S and β-Actin. (**J**) ROS production in control mouse hepatocytes exposed to variable oxygen and temperature stress was measured by flow cytometry. (**K**) Western blots/quantification of HuR, HO-1, p70S6K, and His-H3 expression. For **I** and **K**, the time point 0 hours (+H/R) corresponds to 3 hours of hypoxia followed by 0 hours of reoxygenation. β-Actin served as an internal control for **I** and **K** blots. **H** was analyzed using 2-way ANOVA and Bonferroni multiple comparison test (*n* = 4/group). **I** was analyzed using the Mann-Whitney *U* test (*n* = 3/group). **J** and **K**) were analyzed by 1-way and 2-way ANOVA, followed by Tukey’s HSD test (*n* = 3/group). Data expressed are from at least 3 independent experiments (data are shown as mean ± SEM). *P < 0.05, **P < 0.01 and ****P < < 0.0001. +H/R, hypoxia/reoxygenation.

Previously, we showed that Ceacam1 deficiency activates proapoptotic Phospho-p38 Mitogen-activated protein kinase (p-p38)/ASK1 cell death pathways in cold-stored donor livers (4), while Ceacam1-S–proficient hepatocytes are protected from warm hypoxic stress (5). To determine if oxygen-sensing regulatory pathways under differential temperature stress conditions may regulate the posttranscriptional HuR response to Ceacam1 signaling, we performed in situ hybridization with fluorescent probes designed to hybridize target *Ceacam1* mRNAs at the single-cell resolution level (Figure 2, A–H). Hepatocytes were cultured using an in vitro mimic model of ischemic stress hypoxia/reoxygenation (+H/R) that restricts oxygen levels (hypoxia; <0.01% O_2_) for 3 hours, followed by reoxygenation (5, 14).

We observed that hepatocytes isolated from HuR-deficient livers expressed significantly less *Ceacam1* mRNA compared with Cntl*^fl^* cells under resting state (Figure 2, B, E, and H; *P* = 0.001). In warm-stressed hepatocytes, we observed substantial amplification of *Ceacam1* mRNA in Cntl*^fl^* but not HuR-null cells (Figure 2, C, F, and H; *P* < 0.0001). Cold H/R stress showed appreciably lower *Ceacam1* mRNA levels in HuR-null cells (Figure 2, D, G, and H). Western blots revealed diminished Ceacam1-S protein levels in HuR-deficient hepatocytes cultured under warm H/R, although the data do not reach significance (Figure 2I). We next measured ROS levels in WT cells exposed to H/R under warm versus cold stress using fluorescent H2DCFDA and flow cytometry (Figure 2J). Previously, we showed that Ceacam1-null livers induce high levels of ROS in cold-stored donor livers (4). Here, we observed substantially reduced ROS levels in warm stress compared with mock-treated cells (Figure 2J), consistent with high antioxidant *Ceacam1* mRNA levels observed in Figure 2C. By contrast, cold-stressed cells had significantly higher ROS levels than those in warm stress (Figure 2J, *P* = 0.008). Finally, the low Ceacam1 levels in *Elavl1^fl^* cells correlated with enhanced cellular-stress markers, HO-1 (*P* = 0.01), p70S6K (*P* = 0.045), and His-H3 (*P* < 0.0001) (Figure 2K; *Elavl1^fl^* versus Cntl*^fl^*). Thus, the induction of hepatic Ceacam1 depends on hepatic HuR signaling and provides a mechanistic clue into how antioxidants may homeostatically modulate sterile inflammation in the stressed liver.

### HuR post-transcriptionally targets hypoxia-responsive elements within the Ceacam1 3′UTR necessary for mRNA stabilization.

**Figure 3:**
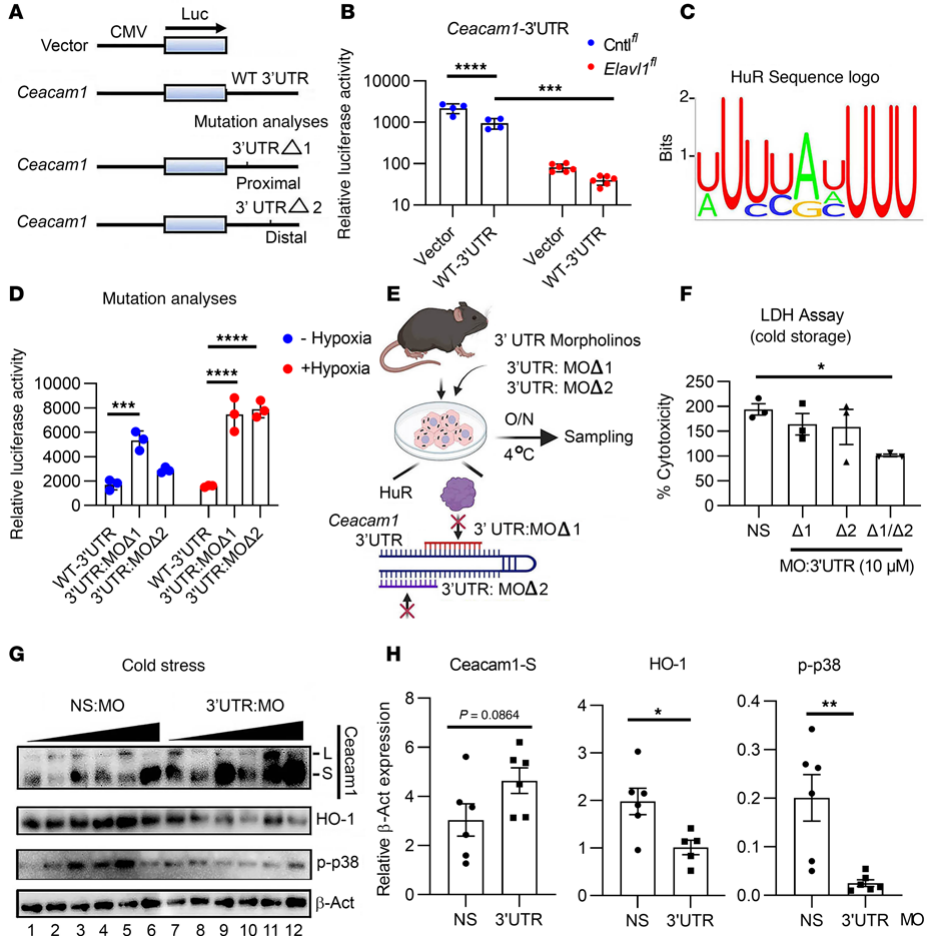
HuR posttranscriptionally targets hypoxia-responsive elements encoded within the *Ceacam1* 3′UTR for mRNA stabilization. (**A**) Luciferase (Luc) constructs showing WT or mutation analyses using 13-nucleotide deletions of the *Ceacam1* 3′UTR sequence (denoted by Δ1 or Δ2). Proximal versus distal refers to the location of the deletion mutations relative to the start of exon 9. CMV refers to cytomegalovirus promoter. (**B**) Cntl*^fl^* hepatocytes (blue) versus *Elavl1^fl^* (red) origin were transfected with Vector or WT-3′UTR sequence (*n* = 4–6/group). Relative luciferase activity was determined as relative light units. (**C**) A HuR Sequence Logo shows conserved nucleotide positions in its RNA binding sequence. (**D**) Cntl*^fl^* hepatocytes were cultured under Hypoxia (blue) versus +Hypoxia (red) conditions using WT versus deletion constructs (*n* = 3/group). (**E**) Schematic of MOs targeting the *Ceacam1* 3′UTR cultured under cold stress. (**F**) LDH cytotoxicity assay in WT hepatocytes cultured with nonspecific (NS) MOs, individual, or combined 3′UTR:MOs, treated with 3-hour cold stress (*n* = 3/group). (**G** and **H**) Western blots and quantitation of Ceacam1, HO-1, p-p38, and β-Actin expression in cold-stressed WT hepatocytes. The concentration of MOs was 2.5 μM (lanes 1–2, 7–8), 5 μM (lanes 3–4, 9–10), and 10 μM (lanes 5–6, 11–12) represented by the ascending black triangle. (**H**) Quantitation of Ceacam1, HO-1, p-p38, and β-Actin expression. **B** and **D** were analyzed using 2-way ANOVA and Tukey’s HSD test. **F** was analyzed by Brown-Forsythe and Welch 2-way ANOVA, followed by a Dunnett T3 post hoc test. **H** was analyzed by unpaired 2-tailed *t* test for 3′UTR:MO versus NS:MO groups (*n* = 6). Data are expressed are from at least 3 independent experiments (data are shown as mean ± SEM). *P < 0.05, **P < 0.01, ***P < 0.001, and ****P < 0.0001.

To determine whether HuR is associated with Ceacam1, luciferase reporter assays were constructed to express either *Ceacam1* WT-3′UTR or vector control sequence (Figure 3A). When constructs were evaluated in Cntl*^fl^* (blue) or *Elavl1^fl^* (red) hepatocytes under normoxia, we noted a significant decline in luciferase activity when HuR was deficient (Figure 3B; *P* = 0.0007*,* WT-3′UTR). A HuR Sequence Logo was then used to screen for binding sites encoded in the *Ceacam1* 3′UTR (Figure 3C). Two candidate 13-nucleotide binding sites were identified in the *Ceacam1* exon 9 sequence, designated proximal 3′UTRΔ1 and distal 3′UTRΔ2. Proximal and distal deletion mutants differed relative to the start of exon 9 (Figure 3A). These deletion constructs were next tested under normoxic (blue) versus hypoxic (red) conditions in Cntl*^fl^* hepatocytes (Figure 3D). We observed a significant increase in luminescence when both proximal 3′UTRΔ1 and distal 3′UTRΔ2 mutations were compared with the control WT Ceacam1 3′UTR sequence (Figure 3D; *P* < 0.0001). These data suggest that hepatic HuR targets multiple responsive elements in the *Ceacam1* 3′UTR during posttranscriptional gene processing.

### MOs targeting the Ceacam1 3′UTR hypoxia-responsive elements overcome transcriptional barriers that repress Ceacam1-S formation under cold stress.

Next, we aimed to determine whether ASOs targeting *cis*-elements at proximal and/or distal regions of the Ceacam1 3′UTR could enhance Ceacam1 protein levels and promote hepatocellular health under cold stress (Figure 3, E–H). To test this, we designed synthetic antisense oligonucleotide MOs to prevent HuR binding to the *Ceacam1* 3′UTR (Figures 3E; 3′UTR:MOΔ1, red; and 3′UTR:MOΔ2, purple). An LDH assay assessed WT hepatocyte health in cells cultured with MOs that block proximal and/or distal hypoxia-responsive elements (Figure 3F). We determined that blocking both hypoxia-responsive elements by MOs was necessary and sufficient to reduce hepatocyte cytotoxicity (Figure 3F; *P* = 0.0479, 3′UTR:MOΔ1/Δ2, hereafter collectively referred to as 3′UTR:MOs, versus nonspecific MO [NS:MO]). Notably, treatment with 3′UTR:MOs restored Ceacam1 expression in cold-stressed WT hepatocytes (Figure 3, G and H), overcoming the previously observed suppression under these conditions (Figure 2, D and G). The rapid translational induction of Ceacam1-S was associated with a significant decrease in the stress-responsive protein HO-1 (*P* = 0.018) and the cell-death marker p-p38 (*P* = 0.005) (Figure 3, G and H; 3′UTR:MO versus NS:MO). Thus, hypoxia-responsive elements encoded in the *Ceacam1* 3′UTR act as a modular platform for hepatic HuR signaling to induce homeostatic healing during cold stress.

### Altered HuR levels by ASOs influence Ceacam1 isoform expression.

**Figure 4:**
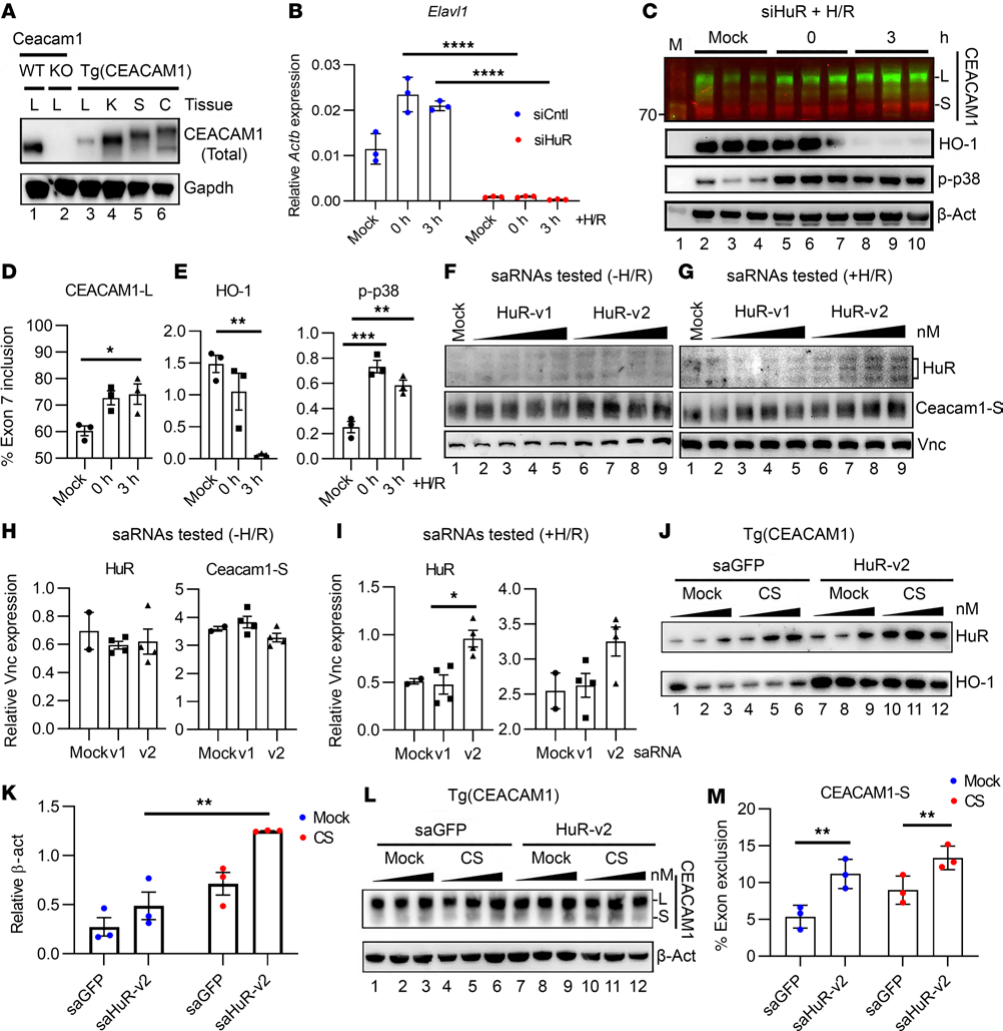
Altered HuR levels by ASOs influence Ceacam1 isoform expression. (**A**) Western blots using antibodies that recognize both mouse and human CEACAM1 were compared in Tg(CEACAM1) hepatocytes expressed in the liver (L), kidney (K), spleen (S), and colon (C). Either isoform may be present (Total). (**B**) qPCR of RNAs isolated from Tg(CEACAM1) hepatocytes pretreated with siRNAs directed to *Elavl1* (siHuR) or a control (siCntl) (*n* = 3/group). (**C**–**E**) Western blots and quantitation of Tg(CEACAM1) hepatocytes treated with siHuR. Antibody 229 was used to detect CEACAM1-L (green), whereas antibody CD66A (red) shows CEACAM1-S expression. **D** was analyzed by the percent inclusion method of (CEACAM1-L)/(CEACAM1-S+CEACAM1-L) levels (*n* = 3), whereas **E** was analyzed relative to β-Actin expression (*n* = 3). (**F**–**I**) Western blot and quantitation of HuR, Ceacam1-S, and Vnc expression in mouse floxed control hepatocytes cultured with saRNAs under normoxia (**F** and **H**) versus hypoxia reoxygenation (H/R; **G** and **I**) conditions. Ascending black triangle denotes increasing concentrations of saRNAs: 15 nM (lanes 2, 6), 30 nM (lanes 3, 7), 60 nM (lanes 4, 8), versus 90 nM (lanes 5, 9). Quantitation for each blot is *n* = 2 mock, *n* = 4/saRNA group. (**J**–**M**) Western blot-assisted HuR, HO-1, Ceacam1-S, and β-Actin expression in Tg(CEACAM1) hepatocytes cultured under cold stress (CS). Quantitation for each blot is *n* = 3/saRNA group. Loading controls were Gapdh (**A**) and β-Actin (**C**, **J**, and **L**), whereas Vnc was used for **F** and **G**. **B**, **K**, and **M** were analyzed by 2-way ANOVA and Tukey’s multiple comparison test, whereas **D**, **E**, **H**, and **I** were analyzed by 1-way ANOVA and Tukey’s HSD test. Data expressed are from at least 3 independent experiments (data are shown as mean ± SEM). *P < 0.05, **P < 0.01, ***P < 0.001, and ****P < 0.0001.

To gain further insight into the regulatory mechanism of Ceacam1 expression, we performed HuR loss-of-function versus gain-of-function studies in a clinically relevant humanized CEACAM1 transgene Tg(CEACAM1) mouse model. Previous studies using Tg(CEACAM1) mice on a WT Ceacam1 background demonstrated that expression of Tg(CEACAM1) serves as an effective model to study how bone marrow granulocytes bind *Neisseria* Opa proteins (19). We crossed global Ceacam1-KO mice with our humanized Tg(CEACAM1) colony to remove cross-reactive interactions with mouse Ceacam1. Next, muCeacam1/huCEACAM1 expression was evaluated in livers (L) from WT, global Ceacam1-KO, and humanized Tg(CEACAM1) mice. Our data show that mouse and human livers expressed 1 isoform of muCeacam1/huCEACAM1, though mouse liver showed better overall expression of the Ceacam1 (Figure 4A, lanes 1 versus 3). Kidney (K) exhibited appreciably higher levels of huCEACAM1, whereas spleen (S) and colon (C) tissues showed extensive evidence of glycosylation (Figures 4A). The distribution of human CEACAM1 in various tissues suggests that humanized CEACAM1-expressing hepatocytes may retain functional similarities to murine hepatocytes expressing endogenous Ceacam1.

Next, we evaluated how RNA interference–mediated loss of HuR signaling in humanized CEACAM1 hepatocytes affects the expression of CEACAM1 isoforms (Figure 4, B–E). siRNAs directed to *Elavl1* mRNA in humanized Tg(CEACAM1) hepatocytes showed significant HuR knockdown (Figure 4B; *P* < 0.0001, *siElavl1* versus siCntl, 3 hours). Loss of HuR promoted increased CEACAM1-L protein expression by Western blots (Figure 4, C and D; *P* = 0.0345, 3 hours versus Mock). Multiplexed fluorescent antibodies detected either CEACAM1-L (green, using antibody α-229) or CEACAM1-S (red) isoforms. Notably, increased CEACAM1-L levels were associated with decreased homeostatic HO-1 expression (Figure 4E; *P* = 0.004, 3 hours versus Mock) and elevated levels of the proapoptotic marker p-p38 (Figure 4E; *P* = 0.005, 3 hours versus Mock).

As a proof of principle, we designed saRNAs to increase HuR expression levels (Figure 4, F–I). SaRNAs are small double-stranded RNA molecules that activate endogenous genes through an RNA-based promoter-targeting mechanism (20). To enhance therapeutic relevance, we screened saRNAs targeting mouse and human HuR promoters to identify a single candidate effective in both species. The saRNA variant 2 (HuR-v2) was less effective at enhancing mouse HuR and Ceacam1-S isoform levels under normoxic conditions (Figure 4, F and H) compared with its effect under hypoxic stress (Figure 4, G and I; *P* = 0.015, HuR-v2 versus HuR-v1). Parallel LDH assays showed improved hepatocyte viability in the presence of saHuR-v2 (60 nM) compared with nonspecific controls (Supplemental Figure 1; supplemental material available online with this article; https://doi.org/10.1172/jci.insight.194227DS1).

When humanized Tg(CEACAM1) hepatocytes were treated with saRNAs targeting either a control promoter (saGFP) or the HuR promoter under cold stress (CS), HuR expression increased by approximately 2.6-fold (calculated as the mean of saHuR 1.25/saGFP 0.49) in response to saHuR-v2 treatment (Figure 4J; *P* = 0.002, Mock versus CS). This corresponded with an increase in HO-1 expression (Figure 4J), along with preferential induction of CEACAM1-S (Figure 4L; *P* = 0.003, saHuR-v2 versus saGFP, CS), reversing the trends seen in RNAi studies (Figure 4, C–E). These findings suggest that hepatic HuR can stabilize muCeacam1/huCEACAM1 expression and that small molecules targeting huCEACAM1 can enhance their protective antioxidant effects in cold-stored livers during the peritransplant period.

### Acute liver injury compromises posttranscriptional HuR regulation of Ceacam1.

**Figure 5:**
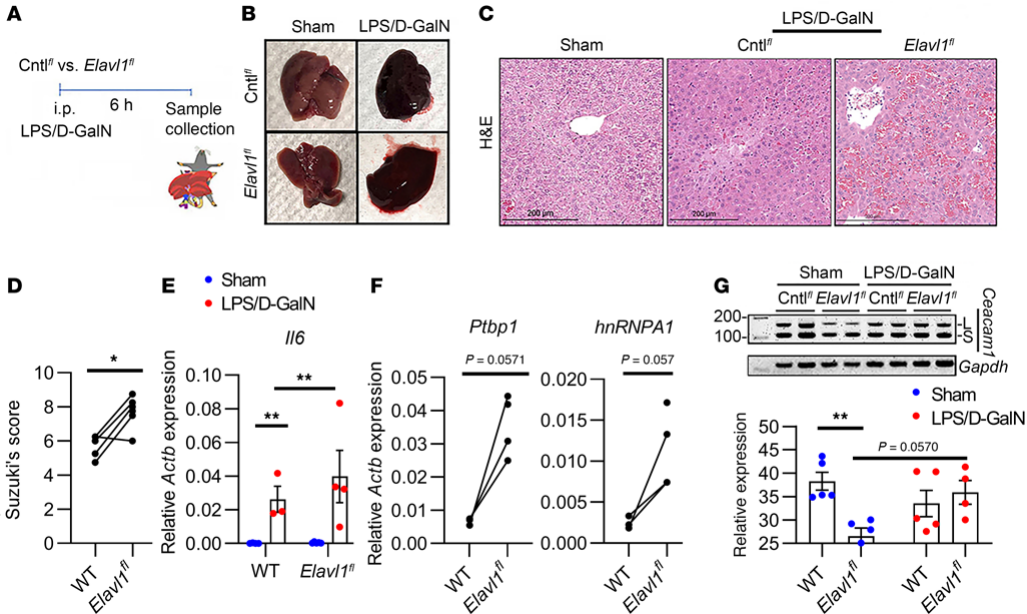
Acute liver injury compromises posttranscriptional HuR regulation of Ceacam1 in vivo. (**A**) Dosing scheme using LPS/D-Galactosamine (LPS/D-GalN) to simulate acute liver injury in mouse livers. Cntl*^fl^* and *Elavl1^fl^* mice were treated 6 hours before liver collection. (**B**) Gross anatomical examination of Sham versus injured livers. (**C**) Representative H&E staining (original magnification, ×10; scaled up 150×). Scale bars: 200 μm. (**D**) Suzuki’s histological grading of acute liver injury tissues (*n* = 5–7/group). (**E** and **F**) qPCR detection of mRNA coding for proinflammatory *Il6* and RNA splicing factors *Ptbp1* and *hnRNPA1*, relative to *Actb* expression (*n* = 3–5/group). (**G**) RT-PCR using exon-junction specific *Ceacam1* primers. Quantitative analyses of percentage of *Ceacam1*-L splice variant (*n* = 4–5/group), relative *GAPDH* expression. **D** and **F** were analyzed by Mann-Whitney *U* analyses; **E** and **G** were analyzed by 2-way ANOVA and Tukey’s HSD test. Data expressed are from at least 3 independent experiments (data are shown as mean ± SEM). *P < 0.05, **P < 0.01.

Next, we aimed to determine whether 3′UTR:MOs targeting hepatic Ceacam1 may potentiate cytoprotective adaptive responses in a clinically relevant in vivo mouse model of sterile liver inflammation. First, hepatic HuR regulation of *Ceacam1* mRNA expression was examined in our LPS/D-Galactosamine (LPS/D-GalN) murine model of acute liver injury (5, 21). This model relies on uridine-5′-triphosphate depletion primarily in the liver by D-GalN to decrease hepatocyte RNA synthesis, leading to excessive ROS and oxidative stress levels, severe hepatic congestion, and rapid cell death. LPS/D-GalN adjuvant was administered to groups of Cntl*^fl^* or *Elavl1^fl^* mice (Figure 5A). At 6 hours, gross examination showed uncontrolled hemorrhaging in the liver from all treatment groups, unlike Sham controls (Figure 5B). Sinusoidal congestion, hepatocellular necrosis, and morphological distortion were more severe in hepatic HuR-deficient livers (Figure 5C), consistent with Suzuki’s liver injury scores (Figure 5D; *P* = 0.014). Moreover, hepatic HuR-deficient livers revealed enhanced *Il6* gene expression compared with Cntl*^fl^* counterparts (Figure 5E; *P* = 0.006, *Elavl1^fl^*, Sham versus LPS/D-GalN). This was accompanied by increased expression of the RNA splicing factors *Ptbp1* and heterogeneous nuclear Ribonucleoprotein A1 (*hnRNPA1*) in *Elavl1^fl^* hepatocytes compared with controls (Figure 5F). Finally, LPS/D-GalN treatment induced a 1.5-fold (calculated as the mean of *Elavl1^fl^* + LPS/D-Gal-N 35.9/Sham 26.5) increase in *Ceacam1-L* mRNA in hepatocyte-deficient HuR mice (Figure 5G; *P* = 0.0571, *Elavl1^fl^* ± LPS/D-GalN). These data demonstrate that HuR shapes hepatocyte responses to injury through a post-transcriptional mechanism that depends on *Ceacam1* alternative splicing during sterile inflammation, linking this process to cellular adhesion and signal transduction.

### MO-mediated targeting of ceacam1 3′UTR alleviates liver inflammation.

**Figure 6:**
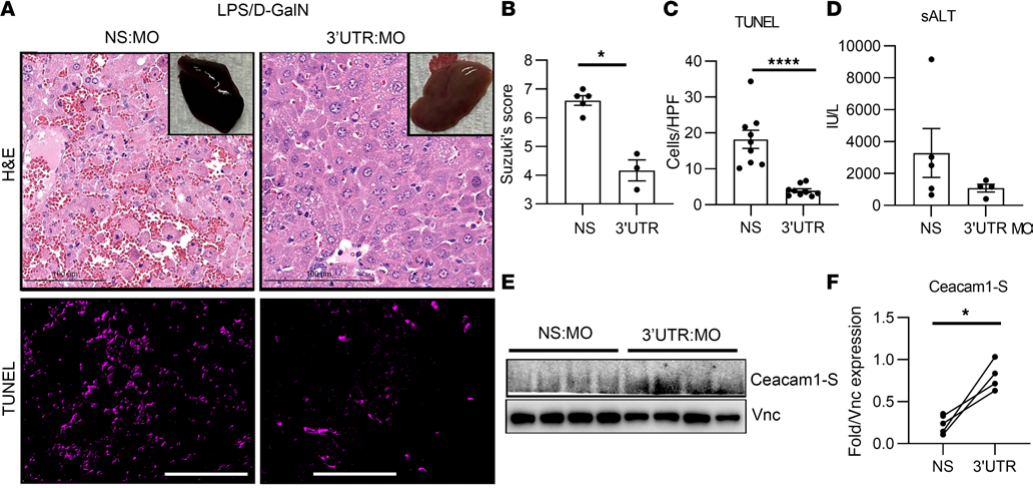
MO-mediated targeting of Ceacam1 3′UTR alleviates liver sterile inflammation. (**A**) Representative examination of WT livers treated with nonspecific (NS) versus 3′UTR:MOs by gross anatomical examination (upper right inset), H&E (original magnification, ×20; scale bars: 100 μm), and TUNEL staining (original magnification, ×40; scale bars: 50 μm, lower right inset). (**B** and **C**) Quantitation of Suzuki’s histological grading (*n* = 4–5/group) and TUNEL staining measured as percent (%) relative frequency (*n* = 3/HPF/ *n* = 9 per group). (**D**) sALT was analyzed using the Kruskal-Wallis test, followed by Dunn’s test (*n* = 4–5/group). IU/liter is international units per liter. (**E** and **F**) Representative Western blot and quantitation of Ceacam1-S, as compared with Vnc expression (*n* = 4–5/group). **B** and **F** were analyzed by a nonparametric Mann-Whitney *U* test, and **C** was analyzed by an unpaired 2-tailed *t* test. Data expressed are from at least 3 independent experiments (data are shown as mean ± SEM). *P < 0.05, ****P < 0.0001.

We next examined whether in vivo stabilization of *Ceacam1-S* using MOs alleviates liver injury. Histological examination revealed that livers treated with 3′UTR:MOs (12.5 mg/kg each) showed significantly improved outcomes compared with controls, as evidenced by gross anatomy (Figure 6A), Suzuki’s scoring (Figure 6B; *P* = 0.04), and TUNEL staining (Figures 6, A and C; *P* < 0.0001). The serum ALT levels further corroborated these findings, showing decreased liver enzyme release in the 3′UTR:MO treatment groups, thus reflecting suppressed hepatocellular damage (Figure 6D). Notably, WT mice treated with the 3′UTR-targeting MOs had significantly higher hepatic Ceacam1-S levels than controls (Figure 6, E and F; *P* = 0.016). These findings highlight the therapeutic potential of 3′UTR-targeting MOs to stabilize Ceacam1-S expression, offering a promising strategy to mitigate liver injury by preserving hepatocyte integrity and function.

### Hepatic CEACAM1 mRNA expression is associated with ELAVL1 mRNA in human OLT.

**Figure 7:**
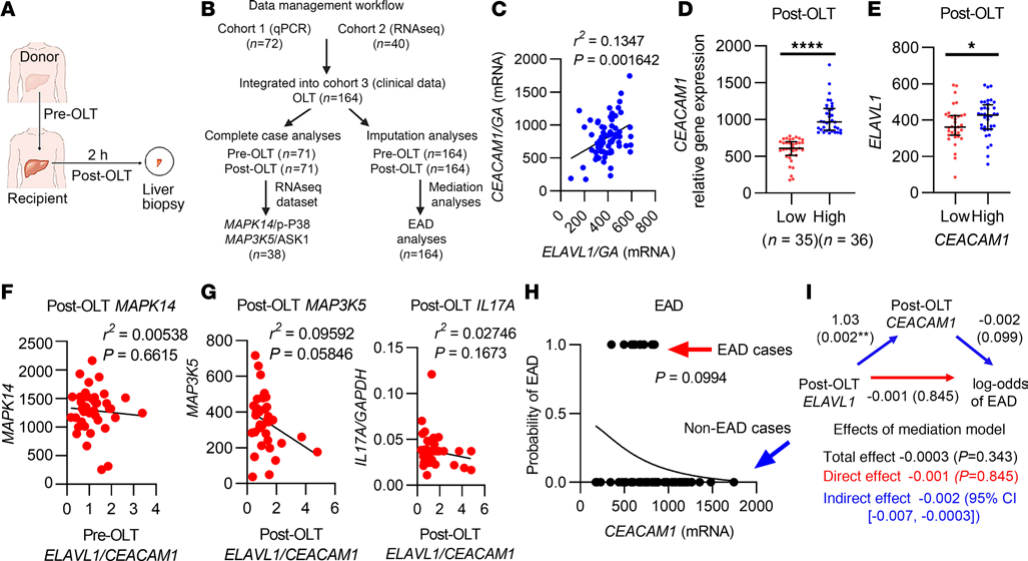
Hepatic *CEACAM1* mRNA expression is associated with *ELAVL1* mRNA levels in human OLT. (**A**) Human liver biopsies were collected before (pre-OLT) and after (post-OLT) transplantation. (**B**) Data workflow for Cohorts 1–3 used for mediation analyses, the regression analyses, and all other clinical analyses. Comparisons between the complete-case mediation analyses and the multiple-imputation mediation analyses established that the complete-case mediation analyses dataset was used for linear regression analyses. (**C**) Correlation of post-OLT *ELAVL1/GAPDH (GA)* versus *CEACAM1/GA* mRNA expression. (**D**) Post-OLT human liver biopsies were divided into low (*n* = 35, red) and high (*n* = 36, blue) *CEACAM1* expression groups. (**E**) Post-OLT *CEACAM1* compared with *ELAVL1* expression. (**F**) Correlation of pre-OLT *ELAVL1/CEACAM1* to post-OLT proinflammatory MAPK14. (**G**) Correlation of post-OLT *ELAVL1/CEACAM1* to post-OLT *MAP3K5* and *IL17A*. (**H**) Logistic regression model with *CEACAM1* predicting the incidence of EAD, controlling for *ELAVL1*. (**I**) The relationship between *ELAVL1* and *CEACAM1* mediates lower log odds of EAD. **C**, **F**, and **G** were analyzed by *t* tests of the linear regression coefficients. (**D** and **E**) were analyzed by nonparametric Wilcoxon Rank Sum tests. (**H**) The *P* value comes from a *z*-test of the logistic regression model coefficient. *P < 0.05, **P < 0.01, ****P < 0.0001 (data are shown as mean ± SEM).

In the clinical arm of the present study, we examined the relationship between *ELAVL1/CEACAM1* in human OLT biopsies (Figure 7A) with a comprehensive dataset of clinical parameters from 164 patients (72 with quantitative PCR [qPCR] and 40 with RNA-Seq measurements; Figure 7B). We found a positive correlation (blue) that was statistically significant between hepatic post-OLT *ELAVL1* and *CEACAM1* mRNAs (Figure 7C; *P* = 0.002). When OLT biopsies were assigned to low (red) versus high (blue) *CEACAM1* expression groups, according to the median split method (cut-off, 813.15) (Figure 7D), once again, significantly higher hepatic *ELAVL1* mRNA levels were found in the high *CEACAM1* biopsies (Figure 7E; *P* *=* 0.04).

There was no significant correlation between *ELAVL1/CEACAM1* grouping and donor data (Supplemental Table 1A), including age, sex, race, BMI, pretransplant serum alanine aminotransferase (sALT), bilirubin, or warm ischemia time. We also found no significant correlation between *ELAVL1/CEACAM1* and the recipient/surgical parameters, including age, sex, race, model for end-stage liver disease (MELD) score, pretransplant blood and liver enzyme tests, bilirubin, prothrombin, and cold ischemia time (Supplemental Table 1B).

To explore the relationships between *ELAVL1/CEACAM1* and proinflammatory profile, we fit linear models using the pre- and post-OLT measurements of *ELAVL1/CEACAM1* to predict the negative (red) after OLT expression of *TIMP1*, *MCP1*, *CXCL10*, *IL17A*, *MAPK14*, and *MAP3K5* (Supplemental Table 2). Indeed, the pre-OLT *ELAVL1/CEACAM1* ratio represented a significant predictor of *MCP1* (*P* = 0.00038), with *MCP1* increasing as the *ELAVL1/CEACAM1* ratio increased. The post-OLT *ELAVL1/CEACAM1* ratio was a significant predictor of *TIMP1* (*P* = 0.0132) and *MCP1* (*P* < 0.0001) expression, with both increasing as *ELAVL1/CEACAM1* increased. Of note, pre-OLT *ELAVL1/CEACAM1* ratio negatively correlated with post-OLT *MAPK14* (protein p-p38) (Figure 7F; *P* = 0.6615) and *MAP3K5* (protein ASK1) (Supplemental Table 2), while post-OLT *ELAVL1/CEACAM1* negatively correlated with *MAP3K5* (*P* = 0.0585) and *IL17A* (*P* = 0.1673) (Figure 7G).

We did not observe a significant association between post-OLT *CEACAM1* expression and the development of EAD after controlling for post-OLT *ELAVL1* levels (*P* = 0.0994). In Figure 7H, the curve was generated with ELAVL1 held constant at its mean value (392.48). The logistic regression model in Figure 7H and the linear regression model in Figure 7C were next used to run a mediation analysis modeling the effect post-ELAVL1 has on EAD incidence through CEACAM1 (Figure 7I). Notably, the indirect effect in this model was significant (95% CI, –0.007 to –0.0003), providing preliminary evidence that post-OLT *ELAVL1*, acting through *CEACAM1*, may decrease the probability of EAD, but more rigorous, structured (and possibly experimental) analysis is needed to confirm this relationship. The direct effect of post-OLT *ELAVL1* on EAD was insignificant (*P* = 0.845). Together, these analyses raise the possibility that the relationship between HuR and CEACAM1 signaling may be important in regulating the hepatic response to inflammation and injury in patients with OLT and that targeted modulation of this pathway may influence clinical outcomes.

### Hepatic ELAVL1 and CEACAM1 mRNA expression correlate with improved tissue homeostasis in discarded human liver biopsies.

**Figure 8:**
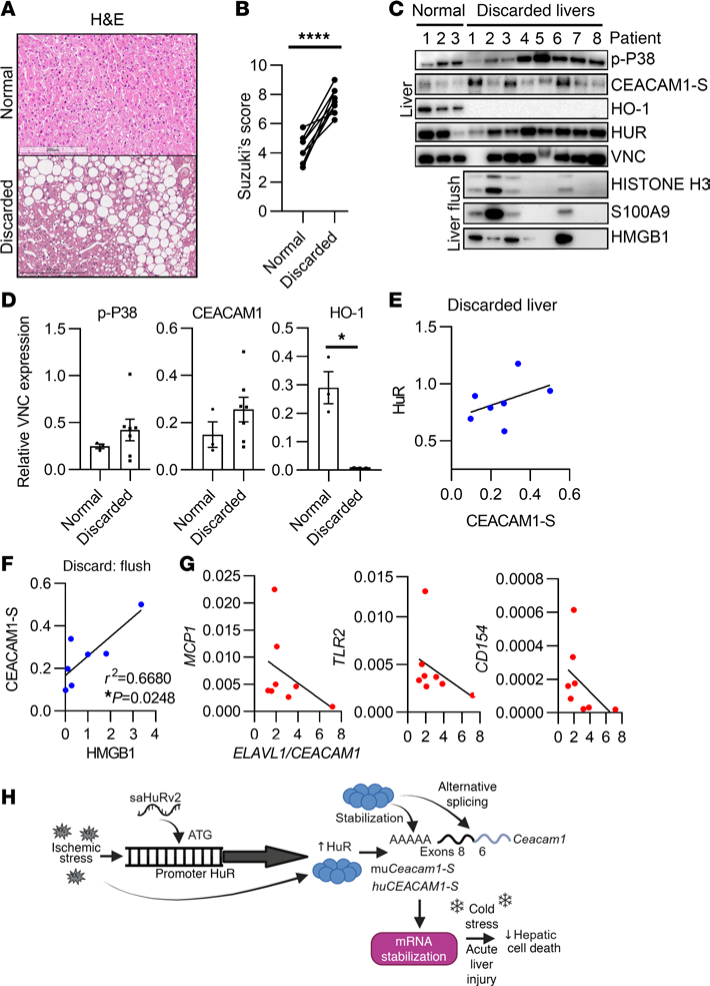
Hepatic *ELAVL1* and *CEACAM1* mRNA expression correlate with improved tissue homeostasis in human discarded liver biopsies. (**A**) Representative examination of H&E-stained discarded livers (original magnification, ×150; scale bars: 100 μm) and TUNEL staining (original magnification, ×150; scale bars: 200 μm). (**B**) Suzuki’s histological grading (*n* = 9/group). (**C**) Western blots assisted detection of markers known to be associated with IRI in normal (*n* = 3), discarded liver tissue, and liver flush samples (*n* = 8/group). The absence of VNC expression led to the exclusion of Patient 1 (discarded) liver tissue from further protein analyses. (**D**) Quantitation of blots shown in **C**, relative VNC (*n* = 3/normal, *n* = 7/discarded). (**E**) Correlation of HUR versus CEACAM1 protein expression in liver tissue (*n* = 7). (**F**) Correlation of CEACAM1-S versus HMGB1 protein expression in discarded liver flush samples (*n* = 8). (**G**) Correlation of discarded liver *ELAVL1/CEACAM1* to proinflammatory markers *MCP1*, *TLR2*, and *CD154* (*n* = 8). (**H**) Model of the relationship of hepatic HuR and Ceacam1-S under high oxygen tension. HuR-targeting saRNAs enhance HuR expression under ischemic stress. This induces HuR coordination with the spliceosome, via so-far uncharacterized mechanisms, to influence the alternative splicing of *Ceacam1*. Additionally, HuR stabilizes *Ceacam1* mRNA during cold stress and acute liver injury, protecting hepatocytes from cell death.

Because of HUR’s posttranscriptional influence on specific target mRNAs and the role that CEACAM1-S alternative splicing plays in driving transcriptional responses and cellular adaptation to cold storage stress, we next aimed to determine whether HUR control of CEACAM1-S expression is essential during cold preservation (4°C) in 8 discarded human liver grafts that were refused due to poor quality. Demographic donor data and organ information are shown in ref. 22. H&E staining showed extensive vacuolization and necrosis as determined by Suzuki’s scoring of liver injury (Figure 8, A and B). Comparison of protein lysates from normal versus discarded livers showed increased levels of p-P38, CEACAM1-S, and HO-1 (Figure 8, C and D). There was no HO-1 expression in discarded livers (*P* = 0.0167) compared with no-injury tissue controls. The protein levels of HUR and CEACAM1-S were positively correlated (Figure 8E), although the association did not reach statistical significance. The levels of High Mobility Group Box 1 (HMGB1), a damage-associated molecular pattern (DAMP), secreted into the liver flush during ex vivo cold storage, highly correlated with CEACAM1 (Figure 8F; *P* < 0.0248). These data led to the hypothesis that HUR may synergize with CEACAM1 to suppress proinflammatory markers of hepatic cell injury and inflammation. Our data show that the *ELAVL1/CEACAM1* axis negatively correlates with *MCP1* (*r* = –0.3333), *TLR2* (*r* = –0.5952), and *CD154* (*r* = –0.5952) gene expression levels (Figure 8G). Finally, Figure 8H presents a model of how Ceacam1-S may undergo fine-tuning in the liver tissue in response to the HuR-stress response.

## Discussion

The present study reports on the posttranscriptional regulatory axis where the RNA-binding protein HuR stabilizes Ceacam1 mRNA to orchestrate isoform-specific dynamics critical for hepatocellular stress adaptation. Although traditionally associated with regulating mRNA stability in cancer (23), HuR controls HO-1–mediated cytoprotection during sterile liver inflammation, and serves as a biomarker of ischemic stress resistance in mouse and human OLT recipients (14). First described in *Drosophila*, the embryonic lethal abnormal visual system (*ELAV*) family of RNA-binding proteins is structurally organized around 3 RNA recognition motifs that enable the recognition of 3′UTR AU-rich elements (ARE) (24, 25). HuR’s 3 RNA recognition motifs, comprised of a βαββαβ structure with 4-stranded antiparallel β-sheet aligned against 2 α-helices, promote the half-life of mRNAs by (a) preventing deadenylation, where the removal of the poly(A) tail leads to mRNA degradation; (b) hindering access of exoribonucleases that promote mRNA decay, such as AU-Rich Element RNA Binding Protein 1 and tristetraprolin; and (c) promoting active export of mRNAs to the cytoplasm, where its translation is accelerated (26–28).

Since our previous study showed the expression of *Ceacam1-S* cycles from a high to a low level in response to acute inflammation and long-term resolution in a mouse model of liver IRI (5) (Figure 1D), we hypothesized here that stabilizing *Ceacam1-S* by activating HuR signaling may favor a long-lasting and sustained hepatoprotection. In situ hybridization studies of floxed control hepatocytes demonstrated robust induction of *Ceacam1* transcripts under warm H/R conditions, consistent with our prior findings that nuclear splicing is essential for cellular adaptation to high oxygen tension (Figure 2C). By contrast, cold ischemic storage of floxed control hepatocytes showed little expression of Ceacam1 (Figure 2D). Hypothermic injury can alter gene expression by suppressing metabolic activity, including RNA polymerase function, inducing chromatin condensation, reducing the accessibility of transcriptional machinery to DNA, and leading to ATP depletion, which causes transcriptional arrest (29). Our previous studies observed that Ceacam1-deficient livers accumulate higher levels of ROS during cold ischemic storage, implicating CEACAM1 in redox homeostasis across both warm and CS contexts (4). Our present study partially corroborated this, as hepatocytes exposed to warm H/R stress significantly reduced ROS levels (Figure 2J). HuR-null hepatocytes also showed substantially less nuclear *Ceacam1* expression under all temperature/oxygen stress conditions tested (Figure 2, E–G), suggesting a putative reprogramming of posttranscriptional splicing and stabilization within the nucleus.

The contribution of HuR to inflammation and disease via alternative splicing regulation remains poorly defined. In a recent study, HuR was indirectly associated with exon skipping events as part of a complex that included Polypyrimidine Tract-Binding Protein 1 (PTBP1) and an N6-Methyladenosine-modified long intergenic noncoding RNA (LINREP) (30). Another study using high-throughput gene ontology shows that HuR is implicated in differentially expressed gene patterns (31). In this study, H/R-treated HuR-null hepatocytes showed increased expression of CEACAM1-L, accompanied by reduced HO-1 and elevated phosphorylated p38, consistent with enhanced apoptotic signaling (Figure 4C). To test the hypothesis that HuR may promote the splicing of Ceacam1-S and to make it clinically relevant, we designed saRNAs to overexpress HuR in mice and humans. Our data establish that mouse HuR stabilizes human *CEACAM1* mRNAs under CS, providing evidence that the human 3′UTR of *CEACAM1* may share sequence or structure similarities with mouse *Ceacam1* that warrant exploitation in future clinical studies (Figure 4, J–M). This is also clinically relevant because OLT injury worsens in cold-stored Ceacam1-deficient donor livers (4). The cross-species regulatory interactions we observed underscore the conserved nature of ARE targeted by HuR. Ongoing research shows significant ARE conservation between humans and mice (32). For example, databases like ARED Organism (http://brp.kfshrc.edu.sa/ARED) integrate ARE-containing transcripts across species, aiding in analyzing how these elements influence mRNA stability, translation, and gene expression (32).

Many reports describe saRNAs as safe, cost-effective, and a well-tolerated means to target undruggable genes to influence disease-relevant gene networks for therapeutic benefit (33). For example, radiological regression of tumors using saRNAs reverse downregulation of CCAAT/Enhancer Binding Protein Alpha (CEBPA) and is effective in patients with advanced hepatocellular carcinoma (34). Another study used saRNAs to differentiate adult human CD34^+^ cells into insulin-secreting cells to treat diabetes (35). Targeting Fms-like tyrosine kinase 1 by saRNAs suppressed proliferation and cell cycle arrest at the G0/G1 phase of human umbilical vascular endothelial cells, causing inhibition of angiogenesis (36). These studies highlight the therapeutic potential of our HuR-targeting saRNAs approach to modulate disease processes driven by dysregulated sterile liver inflammation and hepatocellular injury.

Clinical studies show how ASOs, like AMONDYS 45 (Casimersen), restore reading frame mutations in Duchenne muscular dystrophy (37). In our recent study, splice-blocking MO oligomers targeting *Ceacam1* exon 7 protected murine hepatocytes against temperature-induced stress (5). Surprisingly, the *cis*-elements identified in the Ceacam1-3′UTR were highly responsive to oxygen tension, suggesting that specific molecular dynamics interact with hypoxia-induced regulatory pathways. As a proof of concept, small ASOs were designed to block HuR-targeted Ceacam1-3′UTR *cis*-elements in trans. The aim was to test the hypothesis that MOs may be used as a hepatoprotection-enhancing mechanism in an acute liver injury model (Figure 6). First, our in vitro 3′UTR-MO study showed that targeting these sites effectively enhanced Ceacam1 while simultaneously depressing p38 MAP kinase and HO-1 levels (Figure 3, G and H). In our in vivo study, 3′UTR-targeting MOs significantly attenuated liver injury and enhanced Ceacam1 expression levels (Figure 6). These findings support the therapeutic potential of 3′UTR-targeted interventions to enhance Ceacam1-mediated cellular resilience.

Our clinical data reveal the benefits of *ELAVL1* and *CEACAM1* mRNA expression in patients with OLT. First, we showed that high levels of CEACAM1 were correlated with ELAVL1 and decreased local inflammation (lower proinflammatory biomarkers) in postreperfusion OLT biopsies, which predicted OLT outcomes (Figure 7, F–H, and Supplemental Table 2). Based on our mediation analysis modeling, the effect of post-OLT *ELAVL1* on EAD incidence was significant as a function of post-OLT *CEACAM1* (Figure 7I). This is important, as *CEACAM1* expression was an independent predictor of EAD in human OLT biopsies (4). We also interrogated this relationship by using human liver biopsies discarded for transplantation (Figure 8). CEACAM1-S expression levels were higher in discarded livers, indicative of an active stress-responsive adaptation (Figure 8C). This was not observed for HuR, which is driven primarily by stress-induced subcellular localization rather than overall expression levels. Notably, HO-1 was undetectable in discarded livers. This is clinically relevant, as discarded livers may have progressed beyond the point at which homeostatic HO-1 can exert its protective effects. In future remediation studies of discarded livers, HO-1 may serve as a biomarker for assessing graft quality prior to transplantation.

We observed that the *ELAVL1/CEACAM1* axis was associated with reduced proinflammatory injury signature (e.g., *MCP1*, *TLR2*, *CD154*), suggesting a more immunologically quiescent state that may indicate better functional integrity in otherwise discarded livers. Whether modulating HuR and CEACAM1 during ex vivo perfusion studies may eventually help rejuvenate discarded livers to expand the usable donor pool remains to be determined.

Our study has some limitations. First, the experimental arm relied solely on HuR genetic deletion, which may not fully replicate human physiology, underscoring the need for caution when extrapolating these findings to the human context. Second, using HuR/Ceacam1 boosting small ASOs may result in off-target effects that may confound the interpretation of our mouse and human Ceacam1/CEACAM1 data. Third, the clinical arm involved exploratory analyses, examining variables with both linear regression and mediation models to see which relationships the data supported. Thus, further study will be needed to confirm these relationships. Fourth, well-controlled multicenter clinical trials are needed to bridge this gap because our OLT cohort may not have captured the dynamic range of the HuR/Ceacam1 signaling axis in patients with acute liver injury.

Lastly, we did not study the dynamic crosstalk in the liver microenvironment and the influence of other hepatic cells (e.g., Kupffer, endothelial, and stellate cells). To understand how hepatocytes overexpressing Ceacam1-S influence the course of liver injuries, future studies may benefit from investigating how hepatocyte (parenchymal) Ceacam1 can modulate host (nonparenchymal) immune responses. One possibility is that hepatic Ceacam1 can promote homophilic interactions that spread across the membrane during high oxygen tension conditions. This organized spatial distribution may promote the formation of immune-regulatory clusters, which influence immune checkpoint functions and support immune cell tolerance during liver stress/injury. Indeed, Park et al. (38) recently reported that the homophilic interaction of CEACAM1 attenuated NK cell–mediated killing of CEACAM1^hi^ cancer stem cells in a model of hepatocellular carcinoma (38). Whether these homophilic interactions promote the expression of other immune checkpoint regulators on the hepatocyte surface remains to be determined.

In conclusion, we have identified the hepatic HuR/Ceacam1 signaling axis’s regulatory function, which controls Ceacam1’s response to sterile liver inflammation. Our results provide a foundation for understanding HuR as a potential determinant of donor tissue quality in OLT recipients, with significant implications for future disease modulation and treatment strategies.

## Methods

Supplemental Methods are available online with this article.

### Sex as a biological variable.

Our study examined male mice because of well-established literature and our own preliminary data demonstrating that male mice exhibit more severe liver injury in ischemia-reperfusion injury (IRI) models. This increased sensitivity allows for a more robust assessment of therapeutic interventions. While only 1 sex was studied, the findings are expected to be relevant to both sexes, as the molecular mechanisms investigated are conserved across sexes, though further studies in females are warranted.

### Clinical liver transplant study.

One hundred sixty-four (*n* = 164) adult primary liver transplant patients, recruited under the IRB protocol (13-000143; May 10, 2013, to April 6, 2015), provided informed consent before participation in the study. The recipient and donor variables of the clinical cohort are shown (Supplemental Table 1, A and B). Donor livers, procured from donation after brain death or cardiac death with standardized techniques, were perfused with and stored in cold University of Wisconsin (UW) solution (ViaSpan; Bristol-Meyers Squibb). Protocol Tru-Cut needle biopsies were obtained from the left lobe after liver cold storage at the back table (before implantation) and intraoperatively at about 2 hours after portal reperfusion (before surgical closing of the abdomen) and snap frozen. Study data were collected and managed using REDCap electronic data capture tools. Recipient blood was collected before and after OLT, and liver function was evaluated by sALT/sAST levels. EAD was defined by the presence of one or more of the following: bilirubin level of ≥ 10 mg/dL on POD7, prothrombin time–International Normalized Ratio (PT-INR) ≥ 1.6 on POD7, or AST/ALT level of > 2000 U/L within the first 7 days (39). Cold ischemia time was defined as the interval from the donor’s perfusion with the preservation solution to the removal of the liver from cold storage. Warm ischemia time was defined as the time from removal from cold storage to establishment of liver graft reperfusion.

In our previous studies (4, 5, 14), we used a subset (*n* = 72 patients) of these data to assess the relationship between *CEACAM1*, *ELAVL1*, and OLT. Of these 72 patients, 12 had pre-OLT gene expression data, 16 had post-OLT data, and 44 had pre- and post-OLT data. To supplement the Cohort 1 dataset, in this study, we used a second set of 40 patients (Cohort 2) for whom RNA-Seq was performed both pre- and post-OLT (40) (Figure 7B). Patients from both Cohorts were matched with cases from a third dataset (Cohort 3) containing up-to-date information on clinical parameters for 164 patients. All 3 cohorts were matched using medical record numbers and 2 other ID variables. Two cases from Cohort 1 and 2 from Cohort 2 could not be matched to cases in Cohort 3, so they were excluded from the final merged dataset. In this final dataset, 71 patients had complete gene expression variables (excluding *MAPK14* and *MAP3K5* measurements, which were taken for 38 patients in Cohort 2). To bolster support for the mediation models fit to complete cases, model-based multiple imputation (with 100 imputed datasets) was performed to address missing data with Blimp version 3.2.7 software, as described elsewhere (41, 42). The mediation models were refitted to the imputed datasets to see if the results matched the complete case results. No donor organs were sourced from executed prisoners or other institutionalized persons.

### Mouse liver sterile inflammation study.

All mouse experiments were approved by the UCLA Animal Research Committee (ARC #1999-094) (43). A multifaceted approach was employed, combining in vitro and in vivo studies, where loss-of-function HuR or Ceacam1 mouse models of sterile liver inflammation were employed to identify hepatic signaling pathways that altered lymphocyte/myeloid cell populations (44). Liver injury was assessed by Suzuki’s scoring (45). Small molecule ASOs comprised of saRNAs and MOs enhanced hepatic HuR and Ceacam1 gene expression pathways. In most cases, the sample size for each experimental group (*n*), detailed in the figure legends, ranged from at least 3–7 ice/hepatocyte samples per group. All in vivo experiments were replicated 3 or more times by 2 experimentalists working independently.

### Statistics.

Raw data for experiments in which *n* < 20 is presented in Supplemental Data File 1. GraphPad Prism 8.0.1 was used for statistical analyses, where SEM, chosen to emphasize the precision of the sample mean as an estimate of the population mean, represents the mean value SD quotient relative to the square root of N. For mouse studies, comparisons between 1 or multiple groups were assessed using a 2-tailed Student’s *t* test and 1- or 2-way ANOVA. The Kruskal-Wallis or Mann-Whitney *U* test was employed as a nonparametric (distribution-free) test. Post hoc analyses were performed using Dunn’s multiple-comparison test or Tukey’s HSD test, provided that test assumptions for normality were satisfied by a Shapiro-Wilk or Kolmogorov-Smirnov test. For human data, once post-OLT CEACAM1 and the ratio post-OLT ELAVL1/post-OLT CEACAM1 were divided into low and high groups via median splits, comparisons were made between groups using Wilcoxon rank-sum test (with normal approximation and continuity correction applied to handle ties) for continuous variables and Fisher’s exact test for categorical variables. Linear regression models were fit with pre- and post-OLT ELAVL1/CEACAM1 predicting post-OLT TIMP1/GAPDH, CCL2/GAPDH, CXCL10/GAPDH, IL17A/GAPDH, MAPK14, and MAP3K5, while logistic regression models were fit to predict early allograft dysfunction. These models were also combined to produce mediation models exploring whether ELAVL1 indirectly affected any other gene expression variables or early allograft dysfunction incidence through CEACAM1. *P* < 0.05 (or, correspondingly, a 95% percentile bootstrap confidence interval [CI] that excludes 0 for the indirect effect) was considered statistically significant. For data presented in Supplemental Table 2, the term Coeff is represented by the estimate, SE is represented by the standard error, the term t is represented by t value, and p is represented by Pr(>|t|). In the logistic regression model, a normal distribution is used instead of a t distribution, and thus, instead of a t value, we get a z value, and the *P* value is based on the normal distribution rather than the t distribution, so we have Pr(>|z|). All human analyses were conducted in R version 4.1.1 (42).

### Study approval.

This study was designed to determine the functional role of hepatic RNA binding protein HuR (Human Antigen R) and integral membrane glycoprotein, CEACAM1 (carcinoembryonic antigen-related cell adhesion molecule 1; CD66a) in hepatic acute liver injury in mice and humans. A power analysis, typically between 80% and 90%, was conducted to determine the minimum sample size, given the expected variability and confidence level. Previous studies were used to determine the effect of sample size. Outliers were tested using Prism statistical tests. Mice were randomly assigned to treatment and control groups for in vivo tests to minimize confounding factors such as health or environmental influences. Experimenters were blinded while scoring histological and IHC data to avoid subjective interpretation.

### Data availability.

All data associated with this study are present in the paper or the Supplemental Materials. Values for all data points in graphs are reported in the Supporting Data Values file.

## Author contributions

Conceptualization was contributed by KN, KJD, and JKW. Methodology: was contributed by BC, MC, and KJD. Investigation was contributed by BC, SY, MW, ZYW, TT, RC, and ASK. Surgical procedures were contributed by FK, BC, TT, and KJD. Clinical data analyses were contributed by TDT, MS, FK, and KJD. Writing of the original draft was contributed by BC, KJD, and JKW. Review and editing were contributed by KJD and JKW. Project administration was contributed by DGF and JKW.

## Supplementary Material

Supplemental data

Unedited blot and gel images

Supporting data values

## Version 1 09/23/2025

Electronic publication
