# Supplementary material for: Posttranscriptional control of hepatic CEACAM1 3′UTR by human antigen R (HuR) mitigates sterile liver inflammation
Source: JCI Insight. 2025 Sep 23;10(18):e194227. doi: 10.1172/jci.insight.194227 (PMC12487867; doi:10.1172/jci.insight.194227)
Supplement: Supplemental data [file jciinsight-10-194227-s213.pdf]

Supplementary Materials, Methods, and Results

Title: Post-Transcriptional Control of Hepatic CEACAM1 3'UTR by Human Antigen R (HuR) Mitigates Sterile Liver Inflammation

Authors: Brian Cheng[1], Tristan D. Tibbe[2], Siyuan Yao[1], Megan Wei[1], Zeriel Wong[1], Taylor R. Torgerson[1], Richard Chiu[1], Aanchal Kasargod[1], Kojiro Nakamura[1], Monica Cappelletti[3], Myung Shin Sim[2], Douglas G. Farmer[1], Fady M. Kaldas[1], Jerzy W. Kupiec-Weglinski[1*†], and Kenneth J. Dery[1*†]

Affiliations:

[1]The Dumont-UCLA Transplantation Center, Department of Surgery, Division of Liver and Pancreas Transplantation; David Geffen School of Medicine at UCLA, Los Angeles, CA, USA.

[2]Department of Medicine Statistics Core, David Geffen School of Medicine at UCLA, Los Angeles, CA, USA.

[3]Department of Pathology and Laboratory Medicine, UCLA Immunogenetics Center, Los Angeles, CA, 90095, USA.

Table of contents

Supplementary Materials and Methods……..………..……......………………………………2
Figure S1…………..…………………..……………………………………………………..................10
Table S1……………………………………………………………………...…………………...............11
Table S2..……………………………………………………………………………….......................13
Table S3..………...…………………………………………………………………….......................15
Table S4..……………………………………………………………………………….......................16

Supplementary Materials and Methods

Sex as a biological variable

Our study examined male mice because of well-established literature and our own preliminary data demonstrating that male mice exhibit more severe liver injury in ischemia-reperfusion injury (IRI) models. This increased sensitivity allows for a more robust assessment of therapeutic interventions. While only one sex was studied, the findings are expected to be relevant to both sexes, as the molecular mechanisms investigated are conserved across sexes, though further studies in females are warranted.

Animals and reagents

C57BL/6J, *Elavl1^{fl}* mice (B6.129-Elavl1tm1Thla/J) and Albumin-Cre (Alb-Cre) recombinase (B6.Cg-Speer6-ps1Tg(Alb-Cre)21Mgn/J) mice were obtained from the Jackson Laboratory. Genetic backcrosses resulted in the hepatocyte-deficient HuRKO (*Elavl1^{fl}*) strain used in this study. Littermates that had the floxed *Elavl1^{fl}* mutation but lacked the Alb-Cre mutation were used as controls (Cntl^{fl}) in our study. C57BL/6J wild-type (WT) mice were used for ROS (Figure 2J), morpholino (Figures 3E-3H, 6), and saRNA studies (Figure 4F). Transgenic mice expressing human CEACAM1 in a WT Ceacam1 background have been described previously (19). This study used humanized CEACAM1 Tg(CEACAM1) backcrossed to global Ceacam1KO. Ceacam1KO and humanized Tg(CEACAM1) mice were gifted from the Shively Group (City of Hope, Duarte, CA, USA). All mice, at 8 weeks of age for experimental studies, were housed in the UCLA animal facility under specific pathogen-free conditions and received humane care according to the criteria outlined in the Guide for the Care and Use of Laboratory Animals (43). NucleoSpin Genomic DNA kit (#740952.50, Machery-Nagel; Dueren,

Germany) was used to monitor the presence of *Elavl1*^{f} loci. The primers are listed in Table S3.

Hepatocyte hypoxia-reoxygenation in warm vs. cold storage conditions

Hepatocytes were incubated in serum-free Dulbecco's modified Eagle's medium (DMEM) before incubation in an Anaerobic Gas Generator chamber (<0.1% O2; Thermo Fisher Scientific) at 37°C (warm stress) or 4°C (cold stress) for 3 h. Following this, cells were reintroduced to oxygen under normoxic conditions (air/5% CO2) at 37°C or 4°C for a period of 24 h. For some experiments, time-points 0 h (+H/R) denote 3 h hypoxia and 0 h reoxygenation.

Liver Injury Mouse Model/Flow Cytometry

Acute liver injury was induced by 6h exposure to Lipopolysaccharide/D-Galactosamine (LPS/D-GalN; 30μg/400mg/kg i.p.), purchased from Invivogen (San Diego, CA, USA) and Millipore Sigma (St. Louis, MO, USA), as described (21). The Sham group underwent the same procedures except for the treatment.

Western blots/LDH agarose isoenzyme assay

To create protein lysates, cells and tissue were lysed in M-PER Mammalian Protein Extraction Reagent and supplemented with Halt Protease and Phosphatase Inhibitor Cocktail (ThermoFisher Scientific, Waltham, MA, USA). Total lysates (40μg/sample) were resolved by SDS-PAGE gel electrophoresis and transferred to PVDF membranes. StarBright Blue 700 goat anti-rabbit IgG or DyLight 800 goat anti-mouse IgG was used as secondary Abs for fluorescent blots or Goat anti-rabbit IgG (H+L) secondary antibody, HRP for chemiluminescent blots (ThermoFisher Scientific, Waltham, MA, USA), and high-end imaging was performed (ChemiDoc MP, BioRad Irvine, CA, USA). Antibody 229 was

used for the visualization of the CEACAM1-L isoform (City of Hope, Duarte, CA, USA). This was compared to commercial antibodies, which detect either isoform. The three housekeeping genes were GAPDH, β-ACT/*Actb,* or Vinculin (Vnc). Antibodies used are presented in Table S4. For the LDH isoenzyme assay, protein lysates (20 µg) were prepared and assayed following manufacturer guidelines (Biomedical Research Service, Buffalo, NY).

RNA interference/RNA activation

SiRNAs targeting *Elavl1* (assay ID s67964, 10-50 nM) and Stealth siRNAs (#12935300, 10-50 nM) were purchased from ThermoFisher (Waltham, MA, USA) and used according to manufacturer recommendations. The Stealth siRNA was used as a nonspecific (NS) targeting control. β-ACT/*Actb* was used as a housekeeping gene control. For the RNA activation targeting studies, we used the methods described previously (5). SaRNAs were designed to target the promoter of *Elavl* using the Santa Cruz Genome Browser (SCGB; RefSeq NM_001419). For nonspecific (NS) controls, saRNAs targeting GFP promoters were utilized with the sequence 5'-GAGGGUGAAGGUGAUGCAA-3'. SaRNAs were diluted to 30-90 nM and tested under cold stress at 4 ℃. RNA sequences were synthesized at Integrated DNA Technologies (San Diego, California, USA).

RT-PCR/Quantitative PCR (qPCR)

Total RNA isolation and preparation of cDNA have been described elsewhere (10). Exon-junction PCR amplification of mouse *Ceacam1* mRNA was performed using isoform-specific PCR using a common exon 6 primer (5′-GCTGGCATCGTGATTGGAGTTGTGG-3′) coupled with exons 6-8 junction primers (5′-AGAGTTGTCAGAAGGAGCCAGATCC-3′) to produce Ceacam1-S or (5′-

AGAGTTGTCAGAAGGAGCCAGATTG-3′) to produce Ceacam1-L DNA (5). The High-Capacity RNA-to-cDNA Kit (ThermoFisher, Waltham, MA, USA) was used with 1μg RNA. Amplification of products proceeded to 28-30 cycles, and the product was diluted 1:2 fold in 1x loading dye. The products were resolved on 2.5% Ultrapure Agarose gels (Invitrogen, San Diego, CA, USA) and visualized by staining with 1:20,000 dilution of Diamond Nucleic Acid Dye (Promega, Madison, WI, USA). Gels were photographed on a ChemiDoc MP (BioRad, Irvine, CA, USA), and intensities were quantified using ImageJ image processing software. The mean percent Ceacam1-S or Ceacam1-L was calculated as (Ceacam1-S/(Ceacam1-L + Ceacam1-S) mRNAs x 100 or (Ceacam1-L/(Ceacam1-L + Ceacam1-S) x100. For qPCR, Taqman assays (Table S3) were mixed with TaqMan Gene Expression Master Mix (#43-690-16, Fisher Scientific, Waltham, MA, USA) using the QuantStudio 4 Real-Time PCR system (ThermoFisher, Waltham, MA, USA).

Morpholino studies

Morpholinos (MOs) were obtained from GeneTools LLC (Philomath, OR, USA) and reconstituted according to the manufacturer's instructions. MOs were designed to target *Ceacam1* exon 9. The nonspecific control (NS:MO) was used in parallel studies. For in vitro studies, mouse hepatocytes were incubated with 2.5-10 μM MOs (final concentration) overnight with 6 μl of Endoporter delivery agent, after which cold stress at 4 ºC was applied. For in vivo studies, a concentration of vivo-MOs (12.5 mg/kg each) was administered two and one day before the acute liver injury model.

Luciferase assay

Mouse Ceacam1 exon 9 (3'UTR) nt sequence was obtained from SCGB using RefSeq NM_001039185. The WT sequence (1000 nt from start codon) was cloned into pLenti-

UTR-Dual-Luciferase Cloning Vector (Applied Biological Materials, Ferndale, WA, USA) using services provided by Genewiz from Azenta Life Sciences (Waltham, MA, USA). Luciferase activity was determined using the Nano-Glo Dual-Luciferase Reporter Assay System (Promega, Madison, WI, USA) following the manufacturer's recommendations. The vector control was used as the negative control. Mouse hepatocytes (Cntl^{fl} and *Elavl1*^{fl}) were incubated with 10 µM MOs (final concentration) overnight, after which cold stress at 4 ºC was applied.

H2DCFDA cellular ROS assay

To detect ROS levels, WT hepatocytes were seeded (4.8 × 10^5 total/well) on 12-well plates overnight. The next day, cells were subjected to ±H/R for 3 h at warm or cold stress temperatures. Cells were washed 1x with PBS before treatment with 5 µM final concentration of H2DCFDA (#D399, Fisher Scientific, Waltham, MA, USA) for 30 min at 37 ℃. Cells were washed with PBS and collected using Trypsin-EDTA (0.25%) and phenol red (Fisher Scientific, Waltham, MA, USA) for a minimum time. The mean fluorescent intensity (MFI) was counted by flow cytometry.

RNAScope Multiplex Fluorescent v2 assay (RNA In-Situ Hybridization)

Cntl^{fl} and *Elavl1*^{fl} hepatocytes were isolated and grown in 8-well Nunc Lab-Tek chamber slides (#12-565-18, ThermoFisher, Waltham, MA, USA) at a concentration of 4 x 10^4 cells/well. Following adequate cell confluency, cells were subjected to hypoxia/reoxygenation (H/R) at 37 ℃, and 4 ℃ cold stress, or Mock treatment. Cells were fixed and prepared in accordance with the Cultured Adherent Cell Sample Preparation technical note (ACDBio; Newark, CA, USA). First, the C1 probe for Ceacam1 (#1246571-C1), here serving as the ZZ probe identified in Figure 1A, was added for 2 h at 40 ℃,

within a humidity control chamber. Slides were stored in 5x saline-sodium citrate buffer overnight, and the procedure was completed according to the manufacturer's instructions. The preamplifier and amplifier, which enhance the signal amplification and sensitivity of the probe to the target, were added next. Finally, the fluorophore label was added before the slides were imaged by the UCLA Translational Pathology Core Laboratory.

Hepatocyte isolation/TUNEL assay/serum biochemistry/liver histology/IRI grading

Primary mouse hepatocytes were isolated by a two-stage collagenase perfusion method (44). Cell death in formalin-fixed paraffin-embedded liver sections (4μm) was detected by the Click-it Plus TUNEL Assay Kits for In Situ Apoptosis Detection Kit (#C10619; ThermoFisher, Waltham, MA, USA). Alexa Fluor 647 was used for visualization with a Keyence BZ-X series microscope at 40x original magnification. Liver histology was outsourced to HistoWiz Inc (Brooklyn, NY, USA), and Suzuki's scores were generated by semi-quantitatively blind counting the number of positive cells in 4 HPF (high-power field)/section, original magnification is x20 or x40 by two independent raters (45). Indicators of hepatocellular injury were analyzed by serum aspartate aminotransferase (AST) and serum ALT (sAST/sALT) concentrations as determined by IDEXX Laboratories (Riverside, CA, USA). Samples (5 μl) were combined with SDS-PAGE sample buffer before protein separation on a 4-12% Bolt Bis-Tris Plus Mini Protein Gels (ThermoFisher, Waltham, MA, USA). Gels were run at 160 mV for 30 min, followed by Western-assisted analyses. The LDH Cytotoxicity Assay Kit (#C2LD-100; BioAssays Sciences, Highland, UT, USA) was used to determine hepatocyte cytotoxicity, following manufacturer recommendations.

Statistics

Raw data for experiments in which n<20 is presented in Data File S1. GraphPad Prism 8.0.1 was used for statistical analyses, where SEM, chosen to emphasize the precision of the sample mean as an estimate of the population mean, represents the mean value SD quotient relative to the square root of N. For mouse studies, comparisons between two or multiple groups were assessed using a two-tailed Student's t-test and one- or two-way analysis of variance (ANOVA). The Kruskal-Wallis or Mann-Whitney U test was employed as a nonparametric (distribution-free) test. Post-hoc analyses were performed using Dunn's multiple comparison test or Tukey's HSD (honest significant difference) test, provided that test assumptions for normality were satisfied by a Shapiro-Wilk or Kolmogorov-Smirnov test. For human data, once post-OLT *CEACAM1* and the ratio post-OLT *ELAVL1*/post-OLT *CEACAM1* were divided into low and high groups via median splits, and comparisons were made between groups using Wilcoxon rank-sum test (with normal approximation and continuity correction applied to handle ties) for continuous variables and Fisher's exact test for categorical variables. Linear regression models were fit with pre- and post-OLT *ELAVL1/CEACAM1* predicting post-OLT *TIMP1/GAPDH*, *CCL2/GAPDH*, *CXCL10/GAPDH, IL17A/GAPDH, MAPK14,* and *MAP3K5*, while logistic regression models were fit to predict early allograft dysfunction. These models were also combined to produce mediation models exploring whether *ELAVL1* indirectly affected any other gene expression variables or early allograft dysfunction incidence through *CEACAM1*. A p-value of <0.05 (or, correspondingly, a 95% percentile bootstrap confidence interval (CI) that excludes 0 for the indirect effect) was considered statistically significant. For data presented in Table S2, the term Coeff

is represented by the estimate, SE is represented by the standard error, the term t is represented by t-value, and *p* is represented by Pr(>|t|). In the logistic regression model, a normal distribution is used instead of a t distribution, and thus, instead of a t-value, we get a z-value, and the *P*-value is based on the normal distribution rather than the t distribution, so we have Pr(>|z|). All human analyses were conducted in R version 4.1.1 (39).

<u>Study approval.</u> This study was designed to determine the functional role of hepatic RNA-binding protein HuR (Human Antigen R) and integral membrane glycoprotein, CEACAM1 (carcinoembryonic antigen-related cell adhesion molecule 1; CD66a) in hepatic acute liver injury in mice and humans. A power analysis, typically between 80-90%, was conducted to determine the minimum sample size given the expected variability and confidence level. Previous studies were used to determine the effect of sample size. Outliers were tested using Prism statistical tests. Mice were randomly assigned to treatment and control groups for in vivo tests to minimize confounding factors such as health or environmental influences. Experimenters were blinded while scoring histological and IHC data to avoid subjective interpretation.

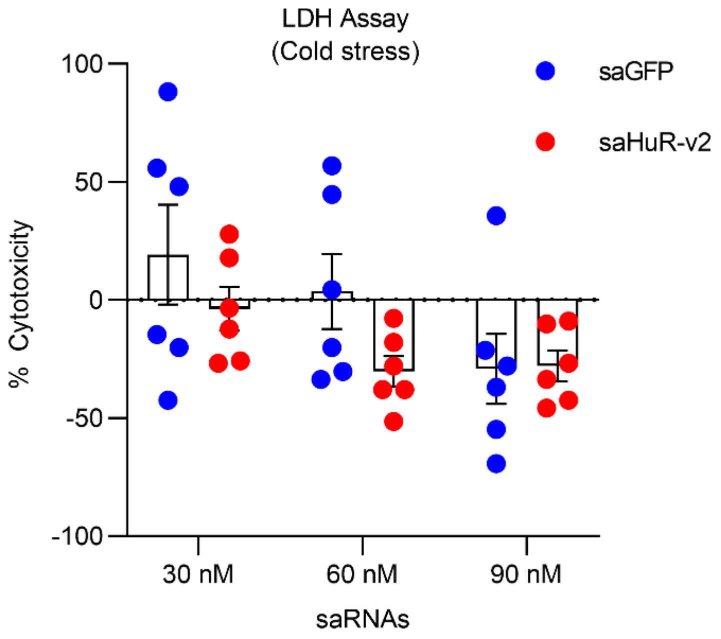

Figure S1. SaRNA optimization by LDH cytotoxicity assay.

Transgenic mouse hepatocytes expressing huCEACAM1 were cultured with different concentrations of saHuR-v2 under cold stress. Sensitivity and specificity were achieved by comparison to control saRNAs targeting the promoter of GFP (saGFP). Quantitation was performed using two-way ANOVA, followed by post-hoc Tukey multiple comparisons test (n=6/group). Data expressed are from at least three independent experiments and represent the mean ± SEM.

Table S1. Demographic data and clinical parameters of human donors and recipients used in the *ELAVL1:CEACAM1* study.

A. Donor parameters*

Variables Count (proportion)	Low Obs. (Mean)	**High Obs. (Mean)	Overall Obs. (Mean)	Test Used	*P* value
Age (years)	35 (37.1)	36 (39.1)	71 (38.1)	571***WRST	0.5007
Gender					
-Male	21 (0.60)	22 (0.61)	43 (0.61)	FET	1.0000
-Female	14 (0.40)	14 (0.39)	28 (0.39)		
Race					
-Asian	5 (0.14)	0 (0)	5 (0.07)	FET	0.0901
-Black	3 (0.09)	3 (0.08)	6 (0.0845)		
-Other	9 (0.26)	15 (0.42)	24 (0.34)		
-White	18 (0.51)	18 (0.50)	36 (0.51)		
BMI (kg/m2)	35 (26.8)	36 (26.1)	71 (26.44)	675-WRST	0.6088
ALT (IU/L)	35 (80.8)	36 (54.1)	71 (67.3)	730-WRST	0.2523
T-Bil (g/dL)	35 (0.96)	36 (0.88)	71 (0.92)	667-WRST	0.6779
WIT (min)	34 (50.4)	36 (56.9)	70 (53.7)	473-WRST	0.1022

*For categorical variables, the count and proportion are provided and, for continuous variables, the number of observations and the mean are provided. **Low and High refer to the Low and High groups formed after median splitting post-OLT HuR/CEACAM1. ***These numbers represent the test statistic. Abbreviations: Alanine Aminotransferase (ALT); Body Mass Index (BMI); Fisher's Exact Test (FET); Observations (Obs.); Total Bilirubin (T-Bil); Warm Ischemia Time (WIT); Wilcoxon Rank Sum Test (WRST)

Table S1. Demographic data and clinical parameters of human donors and recipients used in the *ELAVL1:CEACAM1* study.

B. Recipient parameters*

Variables	Low Obs. (Mean)	**High Obs. (Mean)	Overall Obs. (Mean)	Test Used	*P* value
Age (years)	35 (54.3)	36 (55.8)	71 (55.1)	***569-WRST	0.4825
Gender					
-Male	23 (0.66)	25 (0.69)	28 (0.68)	FET	0.8029
-Female	12 (0.34)	11 (0.31)	23 (0.32)		
Race					
-Asian	2 (0.06)	6 (0.17)	8 (0.11)	FET	0.2603
-Black	3 (0.09)	1 (0.03)	4 (0.06)		
-Other	11 (0.31)	15 (0.42)	26 (0.37)		
-White	19 (0.54)	14 (0.39)	33 (0.46)		
MELD	34 (26.3)	36 (27.7)	70 (27.0)	556-WRST	0.5115
AST (IU/L)	35 (1200)	36 (1057)	71 (1127)	607-WRST	0.7958
ALT (IU/L)	35 (656.8)	36 (564.4)	71 (609.9)	645-WRST	0.8675
T-Bil (g/dL)	35 (8.99)	36 (9.41)	71 (9.20)	709-WRST	0.3666
PT-INR	35 (1.44)	36 (1.46)	71 (1.45)	582-WRST	0.5733
CIT (min)	34 (425)	36 (451.1)	70 (438.5)	495-WRST	0.1710

*For categorical variables, the count and proportion are provided and, for continuous variables, the number of observations and the mean are provided. **Low and High refer to the Low and High groups formed after median splitting post-OLT HuR/CEACAM1. ***These numbers represent the test statistic. Abbreviations: Alanine Aminotransferase (ALT); Aspartate Aminotransferase (AST); Cold Ischemia Time (CIT); Fisher's Exact Test (FET); Model for End-Stage Liver Disease (MELD); Observations (Obs.); Prothrombin Time and International Normalized Ratio (PT-INR); Total Bilirubin (T-Bil); Wilcoxon Rank Sum Test (WRST)

Table S2. Models of pre- and post-OLT *ELAVL1/CEACAM1* intercepts to predict post-OLT outcomes.

	Model	Coeff	SE	t	*P* value
Predictor<br>Outcome	Pre-OLT *ELAVL1/CEACAM1*<br>Post-OLT *MAP3K5*	432.76	48.3	8.96	1.07E-10
Predictor<br>Outcome	Pre-OLT *ELAVL1/CEACAM1*<br>Post-OLT *MAPK14*	1340.88	129.59	10.35	2.48E-12
Predictor<br>Outcome	Pre-OLT *ELAVL1/CEACAM1*<br>Post-OLT *TIMP1*/*GAPDH*	0.06	0.02	2.8	0.0070
Predictor<br>Outcome	Pre-OLT *ELAVL1/CEACAM1*<br>Post-OLT *MCP1*/*GAPDH*	0.11	0.03	3.83	0.0003
Predictor<br>Outcome	Pre-OLT *ELAVL1/CEACAM1*<br>Post-OLT *CXCL10*/*GAPDH*	0.02	0.03	0.88	0.3800
Predictor<br>Outcome	Pre-OLT *ELAVL1/CEACAM1*<br>Post-OLT *IL17A*/*GAPDH*	0.04	0.003	10.21	1.99E-15
Predictor<br>Outcome	Post-OLT *ELAVL1/CEACAM1*<br>Post-OLT *MAP3K5*	416.27	40.42	10.3	2.82E-12
Predictor<br>Outcome	Post-OLT *ELAVL1/CEACAM1*<br>Post-OLT *MAPK14*	1257.04	108.74	11.56	1.13E-13

Predictor<br>Outcome	Post-OLT *ELAVL1/CEACAM1*<br>Post-OLT *TIMP1*/*GAPDH*	0.04	0.02	2.49	0.0200
Predictor<br>Outcome	Post-OLT *ELAVL1/CEACAM1*<br>Post-OLT *MCP1*/*GAPDH*	0.09	0.02	4.8	8.94E-06
Predictor<br>Outcome	Post-OLT *ELAVL1/CEACAM1*<br>Post-OLT *CXCL10*/*GAPDH*	0.02	0.02	1.05	0.3000
Predictor<br>Outcome	Post-OLT *ELAVL1/CEACAM1*<br>Post-OLT *IL17A*/*GAPDH*	0.04	0.003	15.26	8.42E-24

Table S3. Primers used for PCR.

Application	Gene	Primer Duplex Pair
RT-PCR	*AlbCre* (mt)	5’-GAAGCAGAAGCTTAGGAAGATGG-3’
	AlbCre (wt)	5’-TTGGCCCCTTACCATAACTG-3’<br>5’- TGCAAACATCACATGCACAC-3’
	Ceacam1-L	5’-GCTGGCATCGTGATTGGAGTTGTGG-3’<br>5’-AGAGTTGTCAGAAGGAGCCAGATTG-3’
	Ceacam1-S	5’-GCTGGCATCGTGATTGGAGTTGTGG-3’<br>5’-AGAGTTGTCAGAAGGAGCCAGATCC-3’
	Elavl1	5’-TAGGCTCTGGGATGAAACCT-3’<br>5’-CTCTCCAGGCAGATGAGCA-3’
	GAPDH	5’-GTCTCCTCTGACTTCAACAGCG-3’<br>5’-ACCACCCTGTTGCTGTAGCCAA-3’
**Application**	**Gene**	**ThermoFisher Reference Identifier**
qPCR	*CXCL10*	Hs00171042
	hnRNPA1	Mm00446190
	Il1b	Mm00434228
	Il6	Mm01731480
	Mcp1	Mm00441242
	Ptpb1	Mm01303205
	Tnfa	Mm00443258

Table S4. Primary antibodies used for Western blotting.

Ab name	Catalog	Host	Company
α-229	none	Rabbit	Shively Group (City of Hope, Duarte, CA, USA)
β-Act	8457S	rabbit	Cell Signaling
CEACAM1/Ceacam1	14771	rabbit	Cell Signaling
Ceacam1 (Mouse)	MAB6480	rat	R&D Systems
His-H3	4499S	rabbit	Cell Signaling
HO-1	Ab13243	rabbit	Abcam
HuR	SC-5261	mouse	Santa Cruz Biotechnology
HMGB1	6893	rabbit	Cell Signaling
Gapdh	2118T	rabbit	Cell Signaling
p-p38	T180/Y182/7946C	rabbit	Cell Signaling
p70S6K	T389 108D2, 9434T	rabbit	Cell Signaling
PHD1	Ab113077	rabbit	Abcam
S100A9	73425T	rabbit	Cell signaling
Vnc	E1E9V	rabbit	Cell Signaling
