# Supplementary material for: Posttranscriptional control of hepatic CEACAM1 3′UTR by human antigen R (HuR) mitigates sterile liver inflammation
Source: JCI Insight. 2025 Sep 23;10(18):e194227. doi: 10.1172/jci.insight.194227 (PMC12487867; doi:10.1172/jci.insight.194227)
Supplement: Unedited blot and gel images [file jciinsight-10-194227-s214.pdf]

7.15.24 Full unedited gel Fig 1A Genotyping HUR-KO vs WT, Alb Cre PCR

Fig1 - panel A

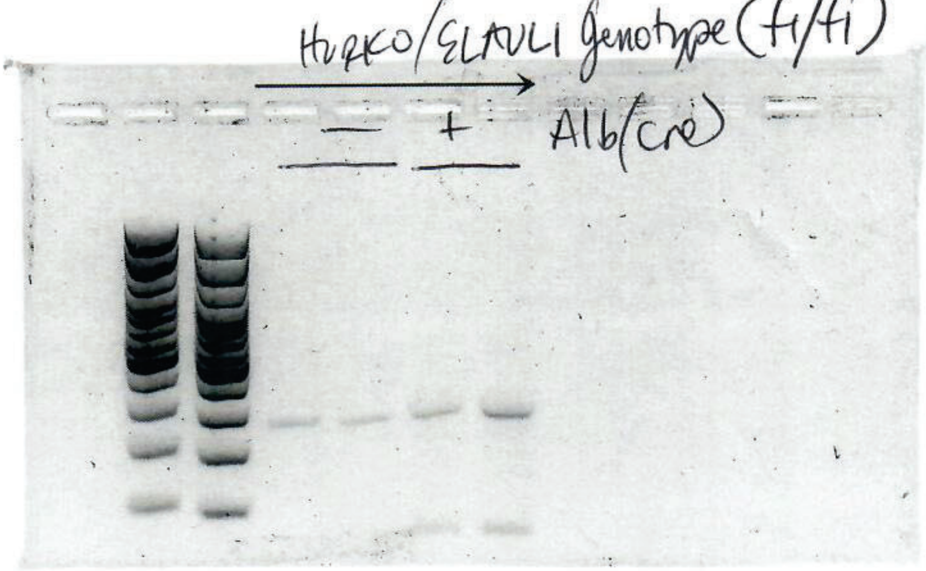

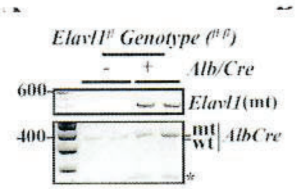

Figure 1

7.11.22 Full unedited gel Genotyping HUR-KO vs WT, HUR PCR

Fig1-
panel
A

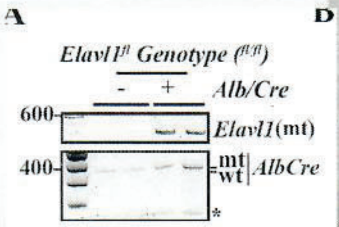

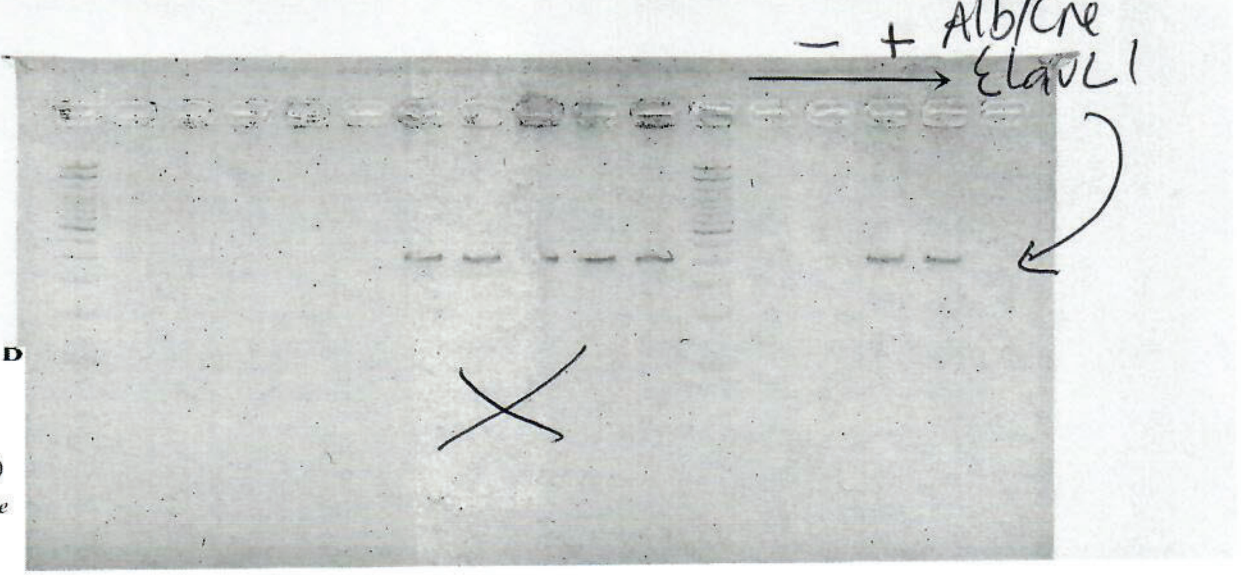

7.15.22 Full unedited gel HUR FINAl, Representative replicate 1 and 2

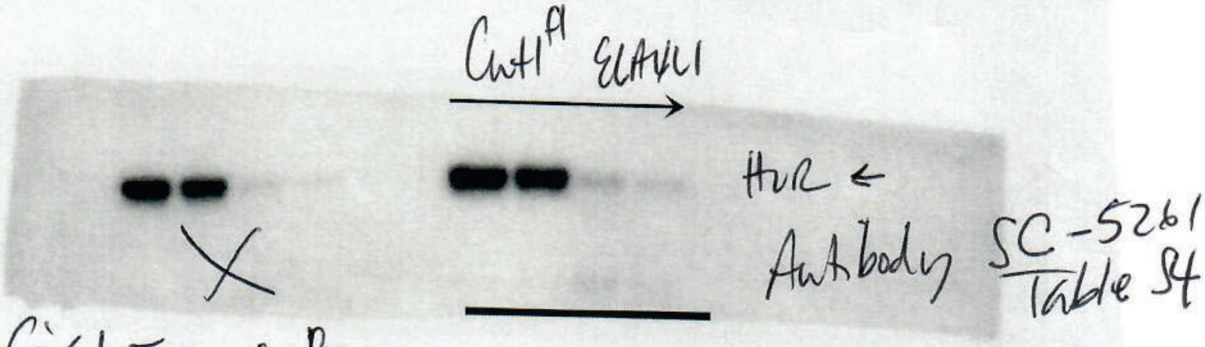

Fig1 - panel B

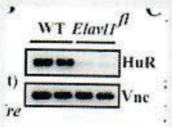

7.15.22 Full unedited gel VNC FINAl, Representative replicate 1 and 2

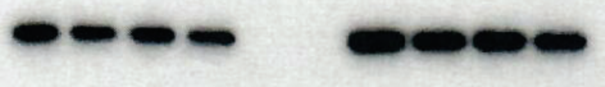

VNC

Antibody E1E 9V
Table S4

X

fig1 – panel B

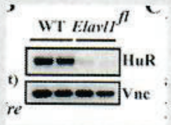

B-5.5.25 Full unedited gel, Crosstalk WT, LysM, CC1KO Hep, LDH Isoenzyme Replicate-1 and Final

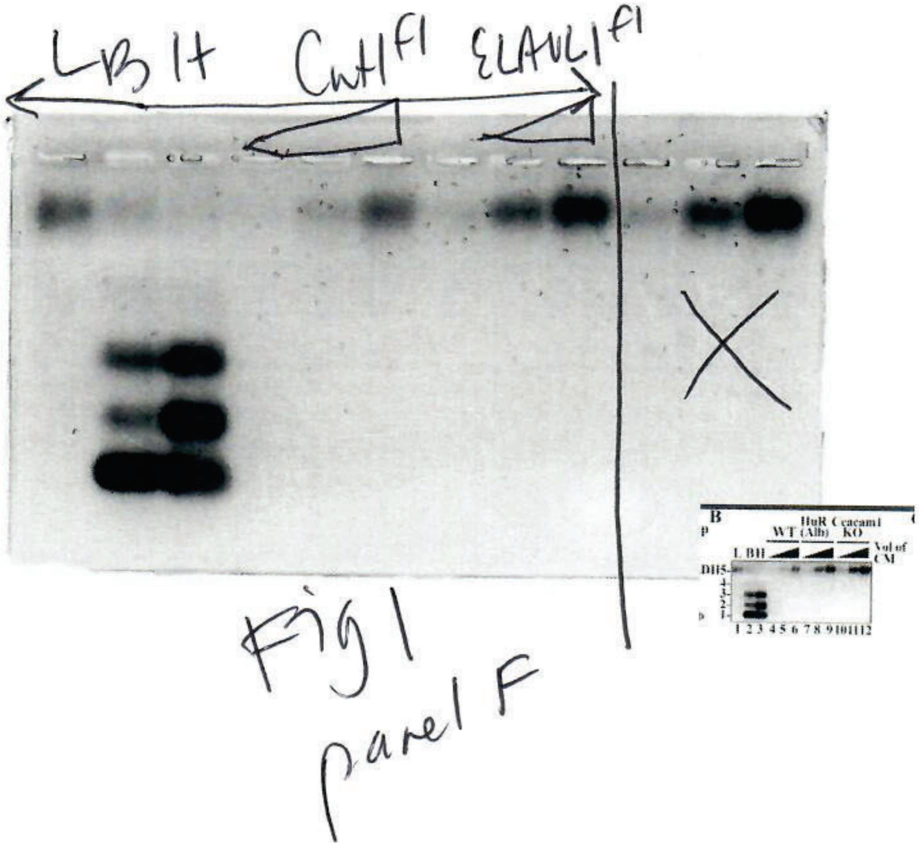

8.12.22 Full unedited gel of HUR-KO CC1-S RAT Ab, Replicate and FINAL

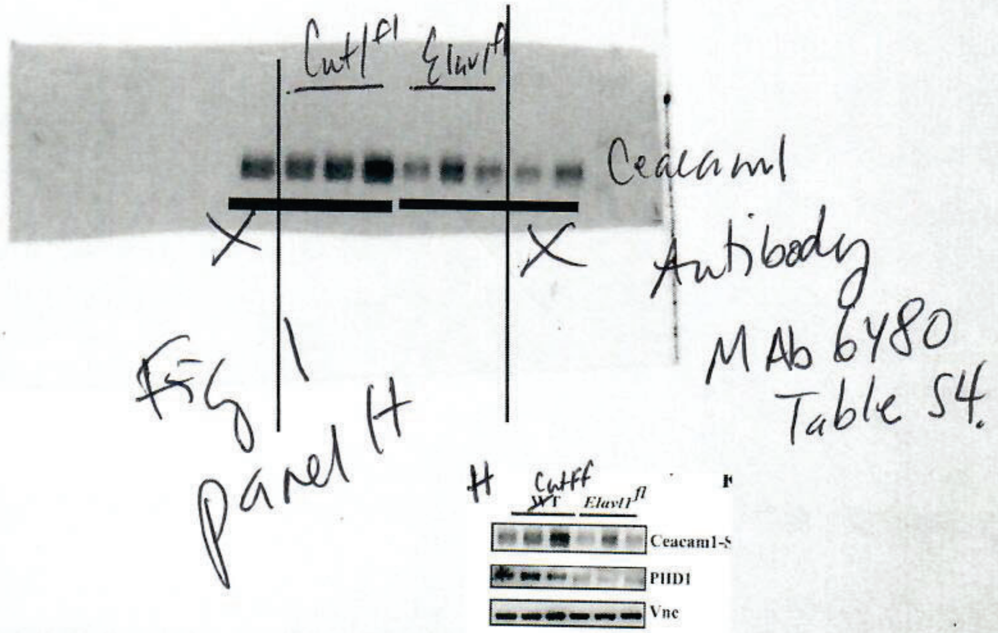

10.17.22 Full unedited gel, anti-PHD1, Replicate and FINAL

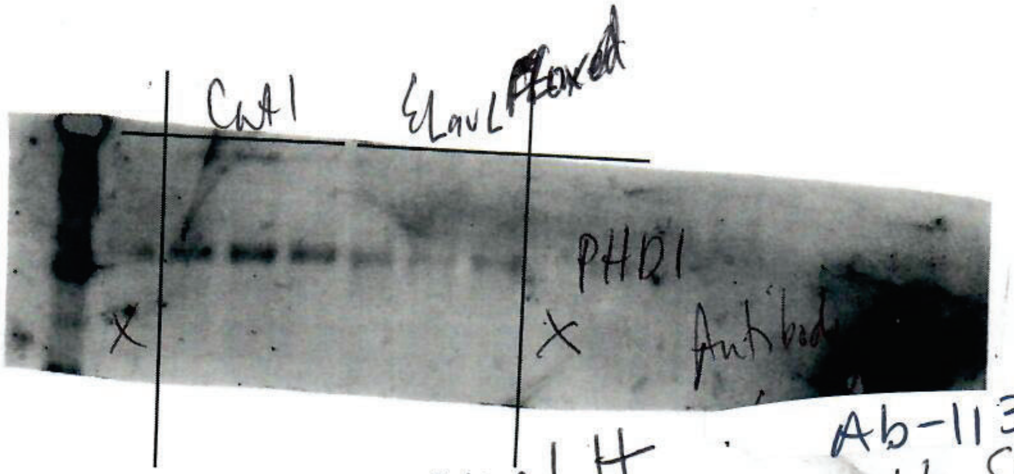

Fig 1 panel H

Ab-113077
Table S4

8.12.22 Full unedited gel of HUR-KO VNC Ab, Replicate and FINAL

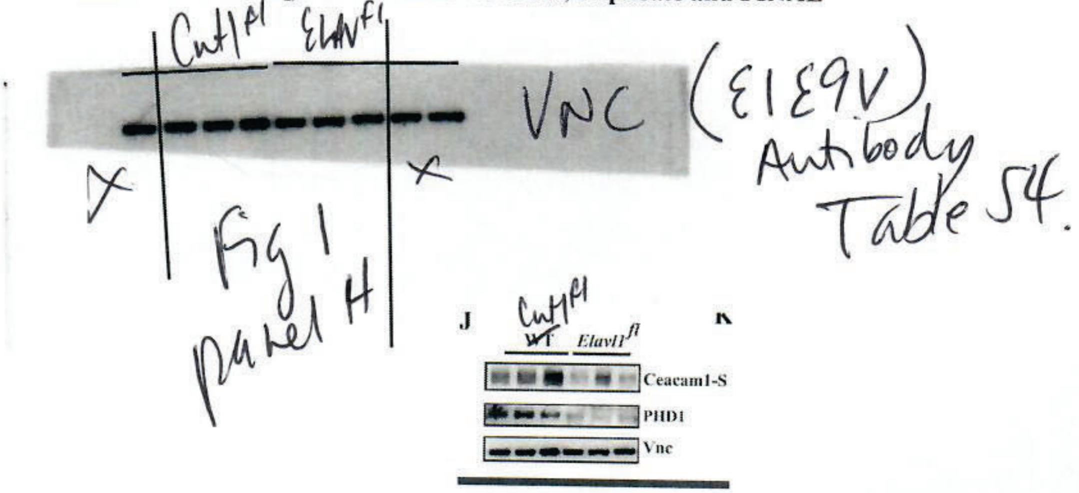

11.19.22 Quantitated on date

Full unedited gel, CC1 in HUR-KO Hepatocytes (Original -107 mouse) FINAL

Cntl fl HuR/ElAvfl

0 3 0 3

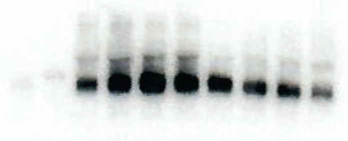

-Ceacam1-S

Antibody MAB6480 Table S4

Fig 2 panel I

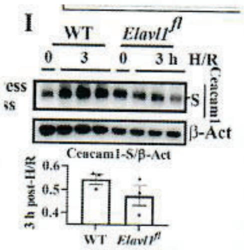

11.19.22 Quantitated on dates

Full unedited gel, BACT in HUR-KO Hepatocytes (Original -107 mouse) FINAL

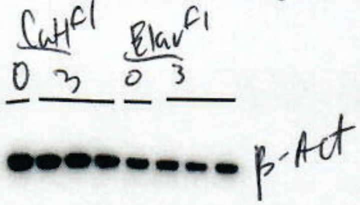

Antibody 84575
Table S4

Fig. 2
panel I

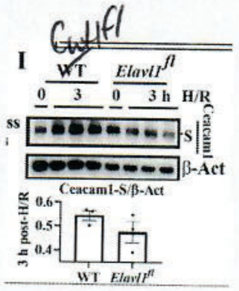

Full unedited gel, HUR in HUR-KO Hepatocytes (Original -107 mouse) FINAL

Cntl fl   Elavl fl

0   3   0   3

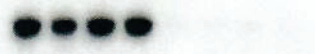

HuR   Antibody SC-5261 Table S4

fig 2, panel K

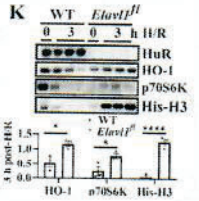

Full unedited gel, HO1 in HUR-KO Hepatocytes (Original -107 mouse) FINAL

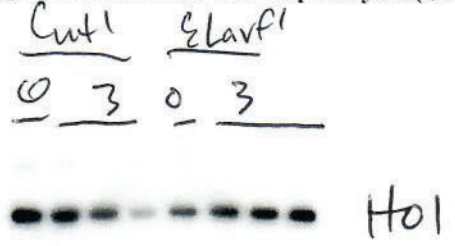

Antibody
GR: 3362818-10
Table S4

Cutfl

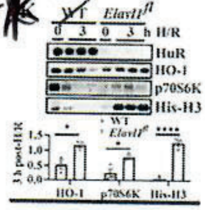

Fig 2, panel K

Full unedited gel, p70Sk in HUR-KO Hepatocytes (Original -107 mouse) FINAL

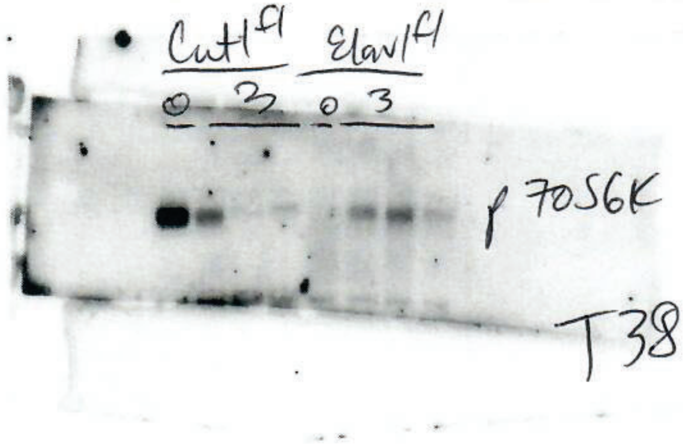

Antibody
T389 10802
9234T
Table S4

Fig 2, panel K

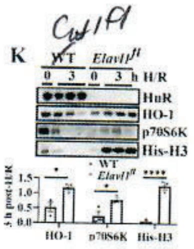

Cul1fl Elavfl

Full unedited gel, HIS-H3 in HUR-KO Hepatocytes (Original -107 mouse) FINAL

0 3 0 3

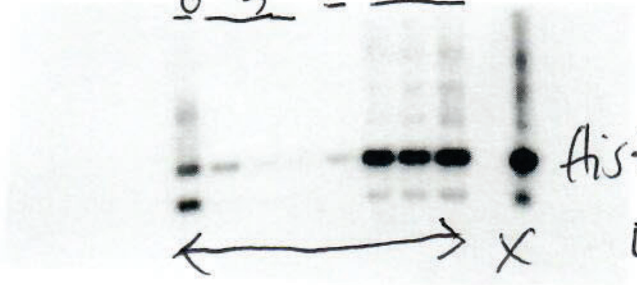

HisH3 Antibody 4499S Table S4

Fig 2, panel K

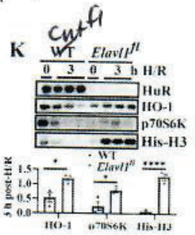

3.25.23 Full unedited gel, WT CC1 morpholinos 3-UTR, mCC1 rabbit Ab Replicate and Final (Darker Version)

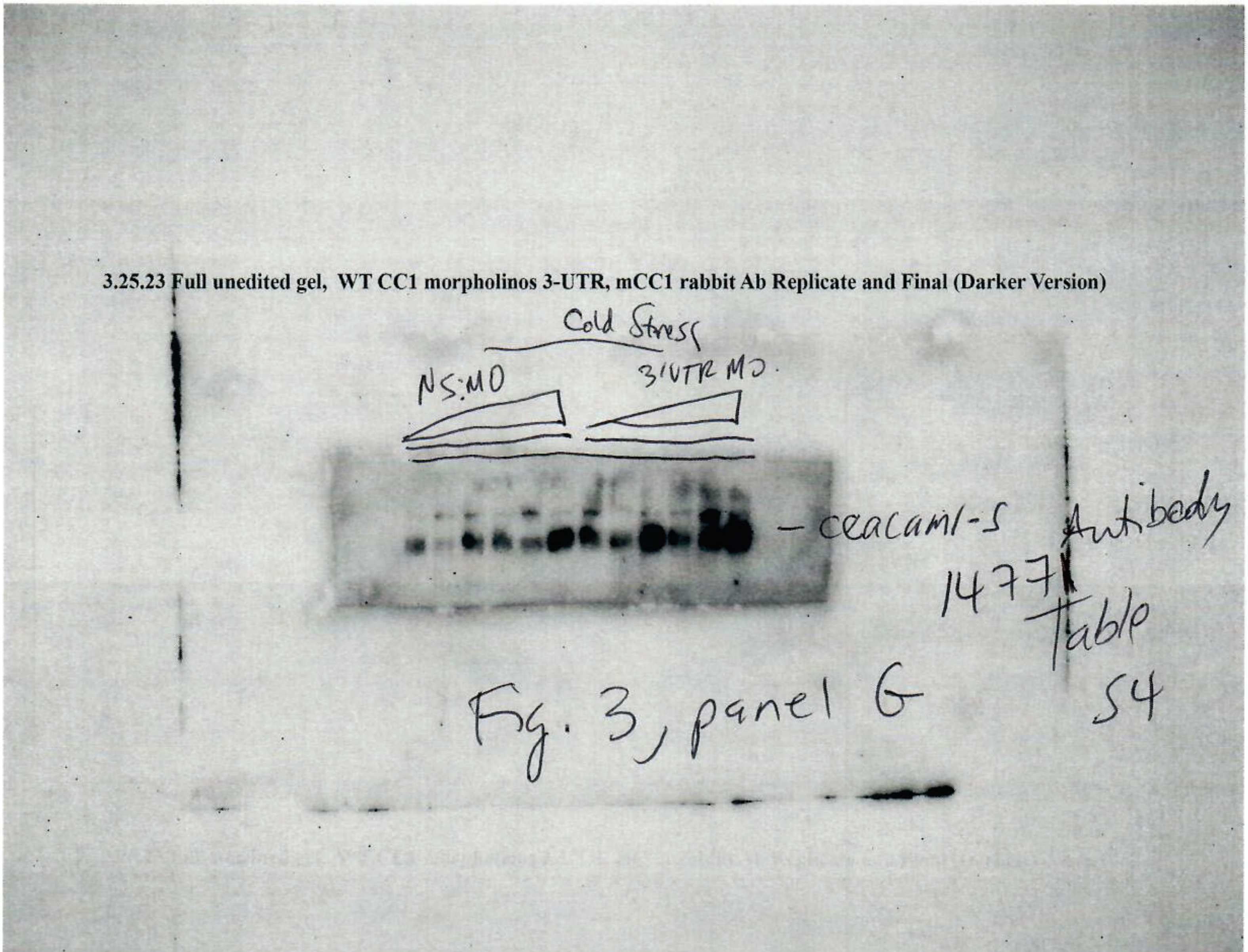

3.28.23 Full unedited gel, WT CC1 morpholinos 3-UTR, HO-1 Ab Replicate and Final

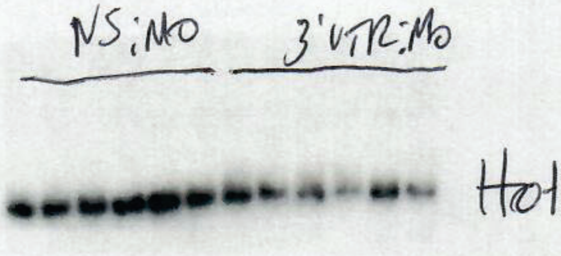

Antibody
Ab13243
Table S4

Fig3, panel B

3.25.23 Full unedited gel, WT CC1 morpholinos 3-UTR, p-p38 Ab Replicate and Final

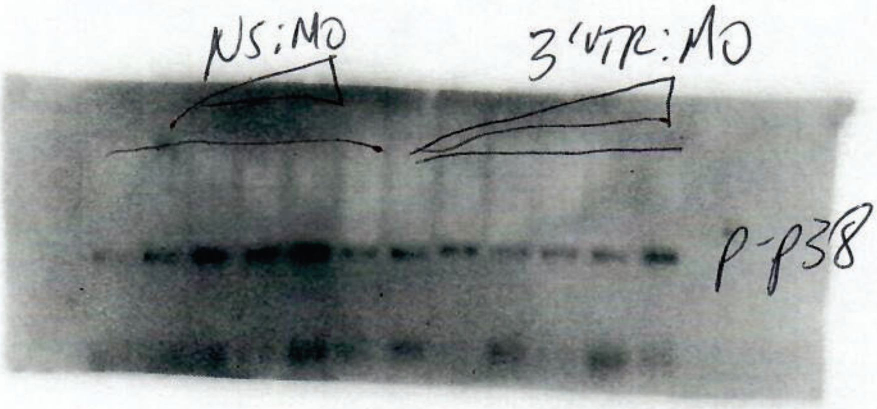

Fig 3, panel G

3.25.23 Full unedited gel, WT CC1 morpholinos 3-UTR, ACTB Ab Replicate and Final

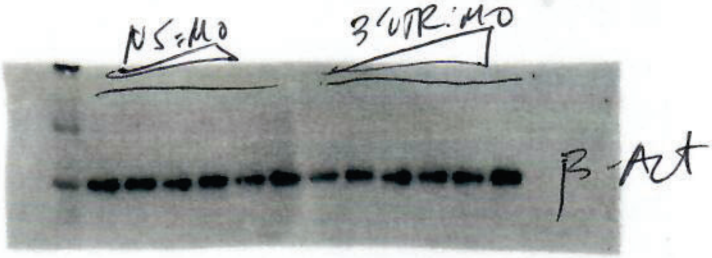

Antibody 84575 Table S4

Fig 3, panel G

A-3.29.25 Full unedited gel, HuTgCC1 vs muCC1 GAPDH Chemi, Replicate and Final

WT Ceacam1 KO Tg Ceacam1

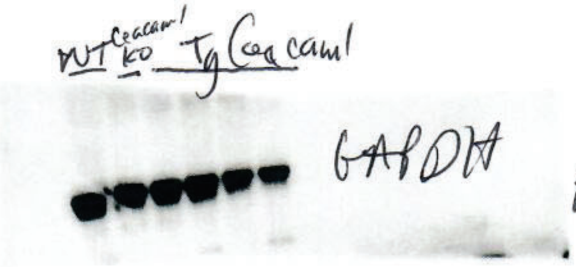

GAPDH

Antibody 2118T
Table 84

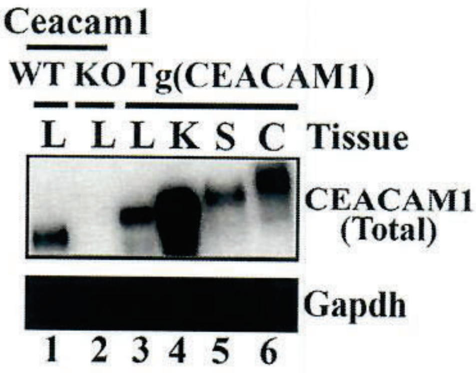

Fig 4, panel A

A-4.1.25 Full unedited gel, muCC1 vs Tg CC1 Naive, Rabbit Ceacam1 Chemi Replicate and Final

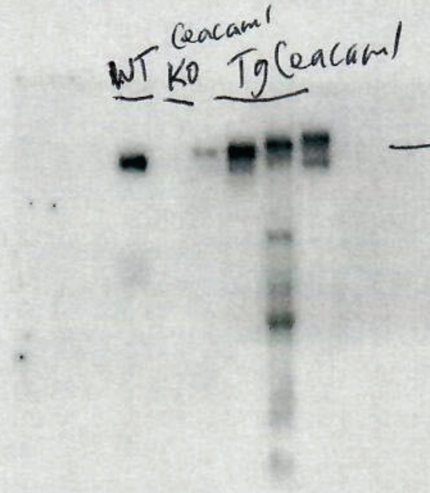

— CEACAM1

Antibody 14771
Table S4

Fig 4, panel A

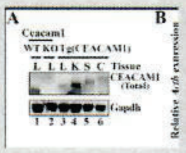

.23 Full unedited gel of huCC1 TG siHuR CC1 HR Timecourse FINAL

siHuR(+H/R)

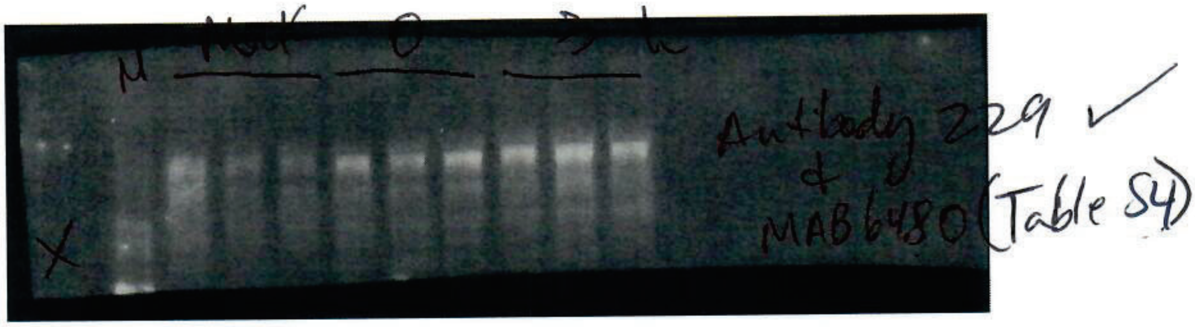

Antibody 229 ✓
&
MAB6480 (Table S4)

Fig 4, panel C

1.20.23 Full unedited gel of huCC1 TG siHuR HO-1 HR Timecourse FINAL

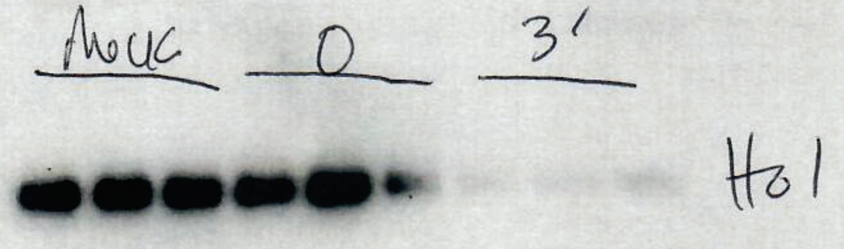

Antibody
Ab13243
Table S4

fig 4, panel C

1.27.23 Full unedited gel of huCC1 Tg +H/R, p38 AB, Replicate and FINAL

Mock 0 3'

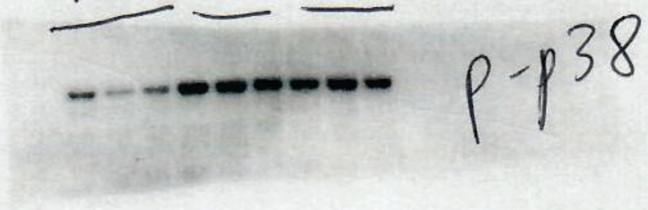

p-p38

Antibody 7946C

See Table S4

Fig 4, panel C

1.18.23 Full unedited gel of huCC1 TG siHuR BACT HR Timecourse F

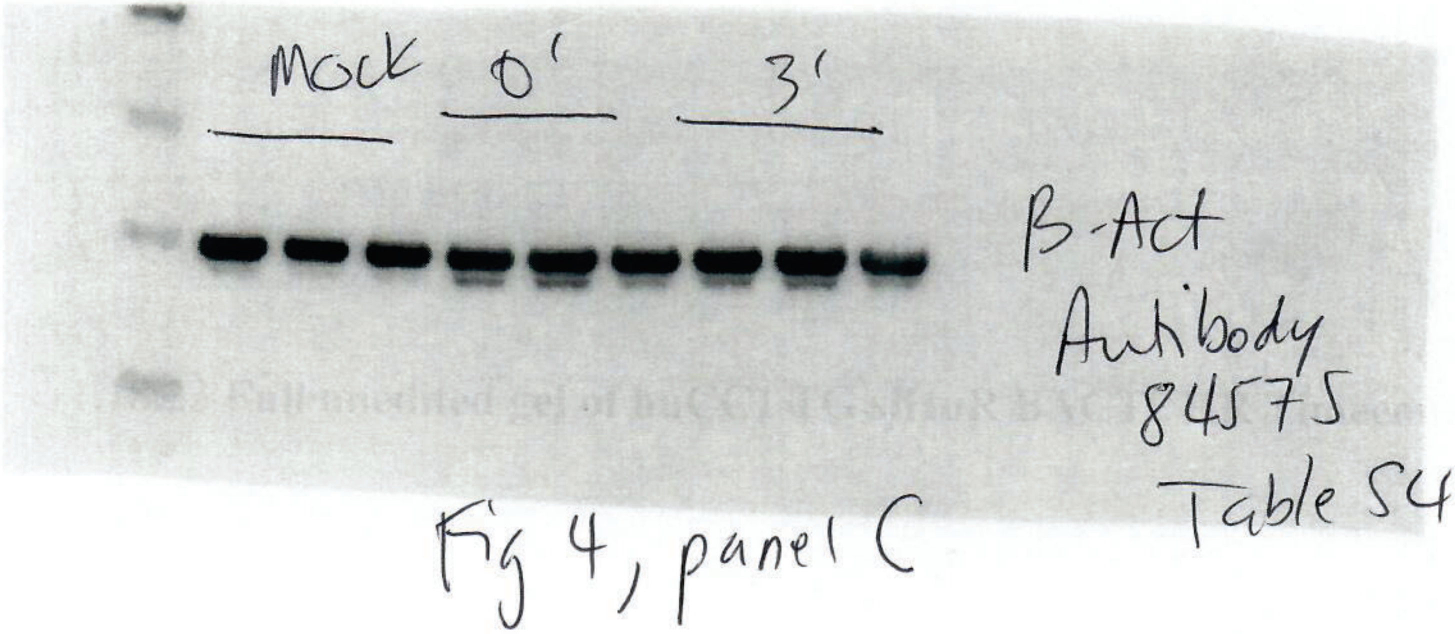

Fig 4, panel C

7.20.23 Full unedited gel FINAL, anti-[illegible]uR, saRNA test No Hypoxia conditions

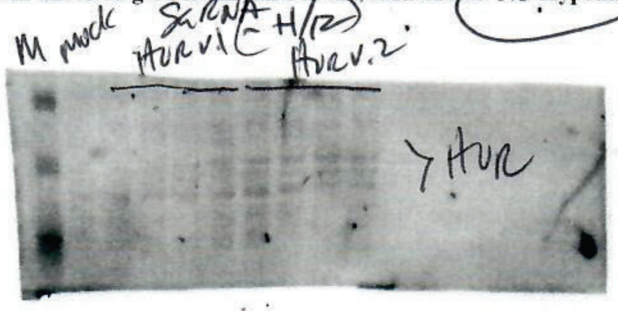

Antibody
SC-5261
Table S4

Fig 4, panel F, Left

7.20.23 Full unedited gel FINAL, anti-m HuR, +saRNA conditions Plus Hypoxia

saRNA (+H/R)

M mock HuR-v1 HuR v2

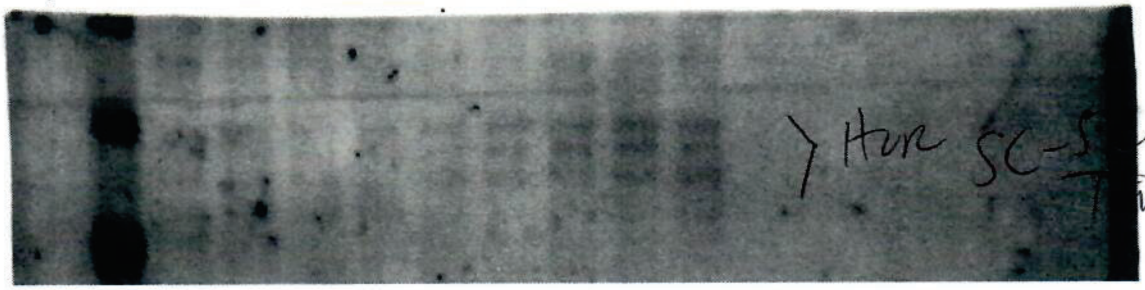

} HuR

Antibody SC-5261 Table S4

Fig 4, panel ~~F~~ G, Right

7.25.23 Full unedited gel FINAL, #2 Replicate anti-rat mCC1, +saRNA conditions NO Hypoxia

saRNA (-H/R)

mock HuR V1 HuR V2

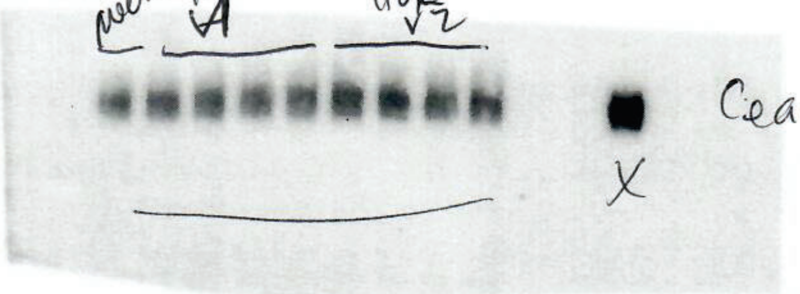

Ceacam1-5

MAB6480
Table S4

Fig 4 panel F, Left

Antibody 229
4

7.25.23 Full unedited gel FINAL, Replicate 3 and anti-rat CC1, +saRNA conditions PLUS Hypoxia

saRNA (+H/R)

Mock H/R v1 H/R v2

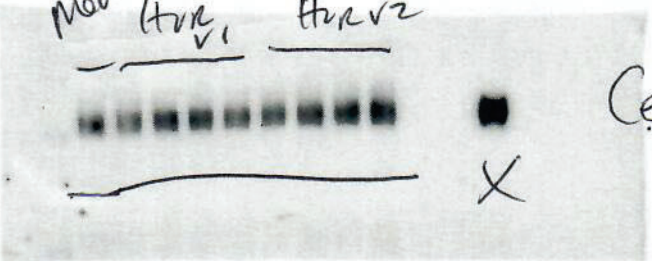

Ceacam1

Table S4 Antibody MAB6488

Fig 4, panel F, Right

7.20.23 Full unedited gel FINAL, anti- VNC , +saRNA conditions NÓ Hypoxia

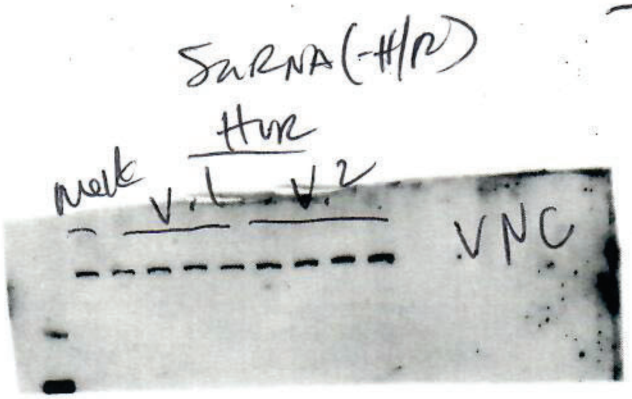

Antibody E1E9V
Table S4

Fig. 4, panel F, Left

saRNA (H/R)

7.20.23 Full unedited gel FINAL, Replicate 3 anti-r VNC, +saRNA conditions Plus Hypoxia

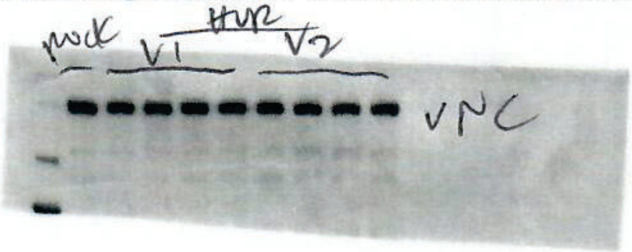

E1E9V
Table S4
Antibody

fig 4, panel ~~F~~, ~~R+~~ G

9.22.23 Full unedited gel of huCC1 TG saRNA v2 CS, HuR rabbit Chemi AB Replicate and FINAL

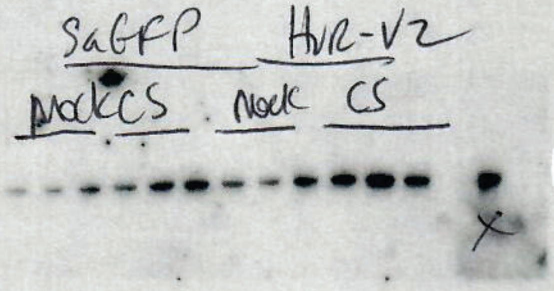

HuR

SC-5261
Table S4

fig 4, panel J

SaGFP HuR v2

9.12.23 Full unedited gel of huCC1 TG HO1-Ab Chemi saRNA CS Replicate and FINAL

mock CS mock CS

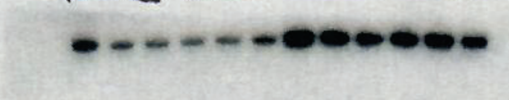

HO-1

Anti-Ab13243

Table S4

Fig. 4, panel ~~G~~

SaGFP SaltvR v2

9.15.23 Full unedited gel of huCC1 TG saRNA v2 CS, rabbit CEACAM1 AB Replicate and FINAL

mock CS mock CS

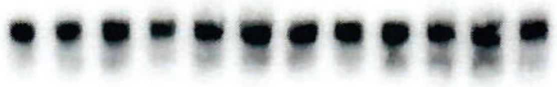

ceacam1-S

α-14771

Table S4

Fig 4, panel ~~A~~

Sa GFP SaHuRV2

9.14.23 Full unedited gel of huCC1 TG saRNA CS, BACT AB Replicate and FINAL

Mock CS Mock CS

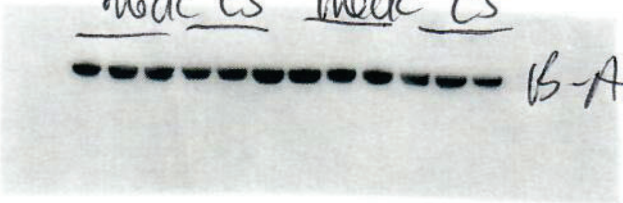

B-Act

Antibody 84575

Table S4

fig 4, panel L

G-8.29.22 Full unedited gel of Fig 2, panel H, RT-PCR CC1-S +DGAL FINAL

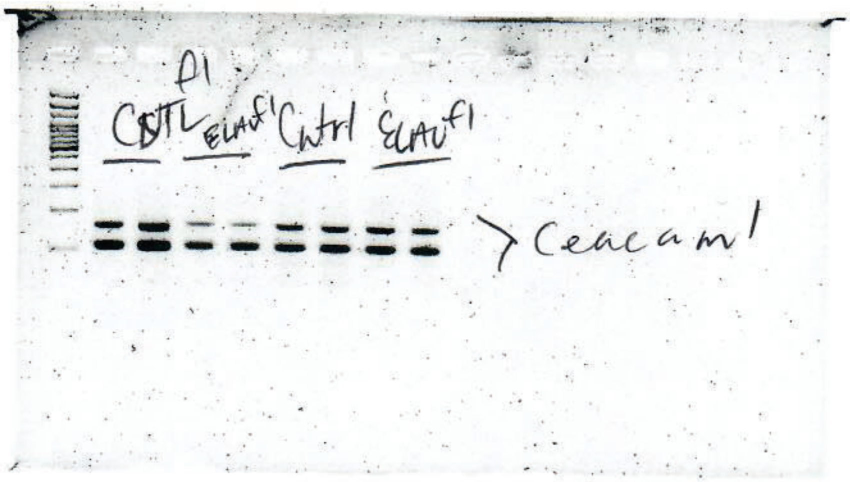

Fig 5, panel G

10.26.22 Full unedited gel of Fig 2, panel H, RT-PCR GAPDH +DGAL

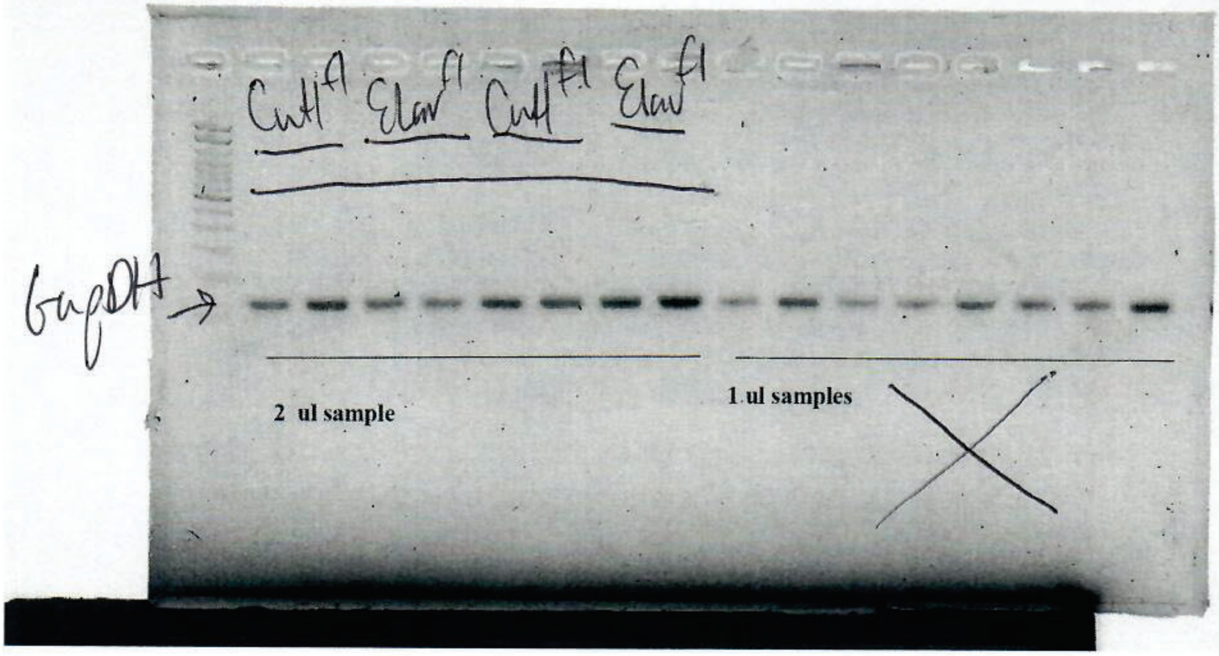

Fig 5, panel G

6.17.24 Full unedited gel, NS vs 3'UTR MO DGAL, Fluor Ab Chemi Replicate and Final

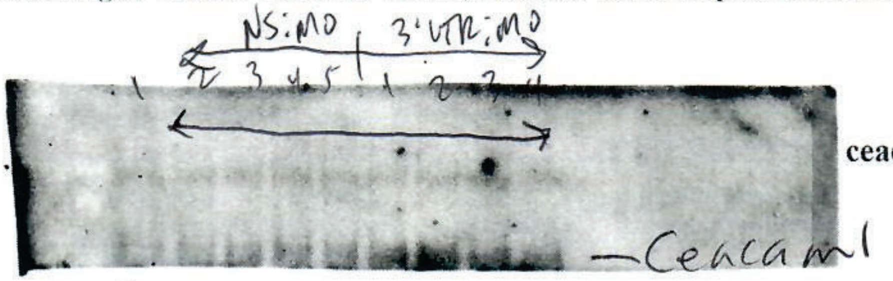

ceacam1 antibody

NSMO n=5 3'UTR n=4

MAB6480
Table S4

Fig 6, panel E

6.17.24 Full unedited gel, NS vs 3'UTR MO DGAL, Vnc Ab Chemi Replicate and Final

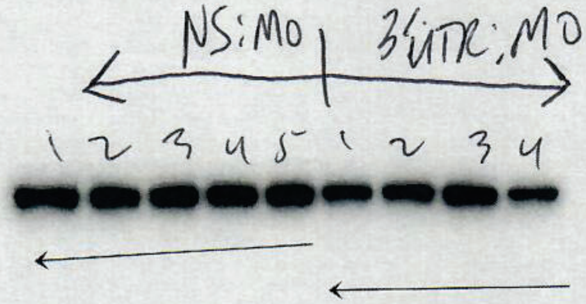

α-E1E9V
Table S4

NSMO n=5 3'UTR =5

Fig 6, panel E

8.17.23 Full unedited gel Fig 1, rabbit p-p38 (Chemi) Ab, Replicate and Final

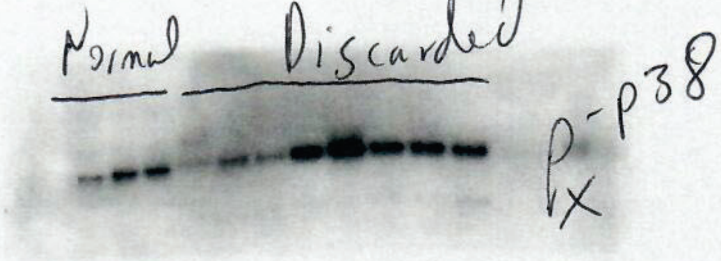

Fig 8, panel C

Anti-
7946C
Table S4

8.16.23 Full unedited gel Fig 1, rabbit human CEACAM1 (Chemi) Ab, Replicate and Final

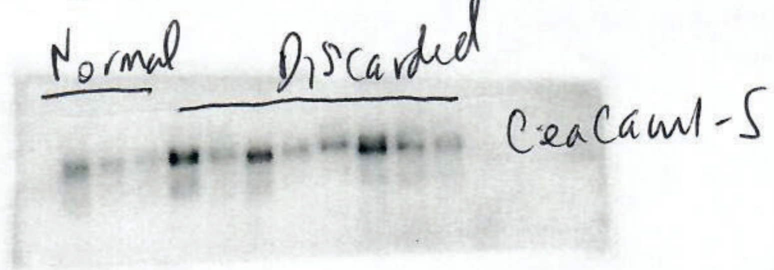

Fig 8, panel C

α-1477/
Table S4

8.17.23 Full unedited gel Fig 1, rabbit HO1 on fl membrane (Chemi) Ab, Replicate and Final

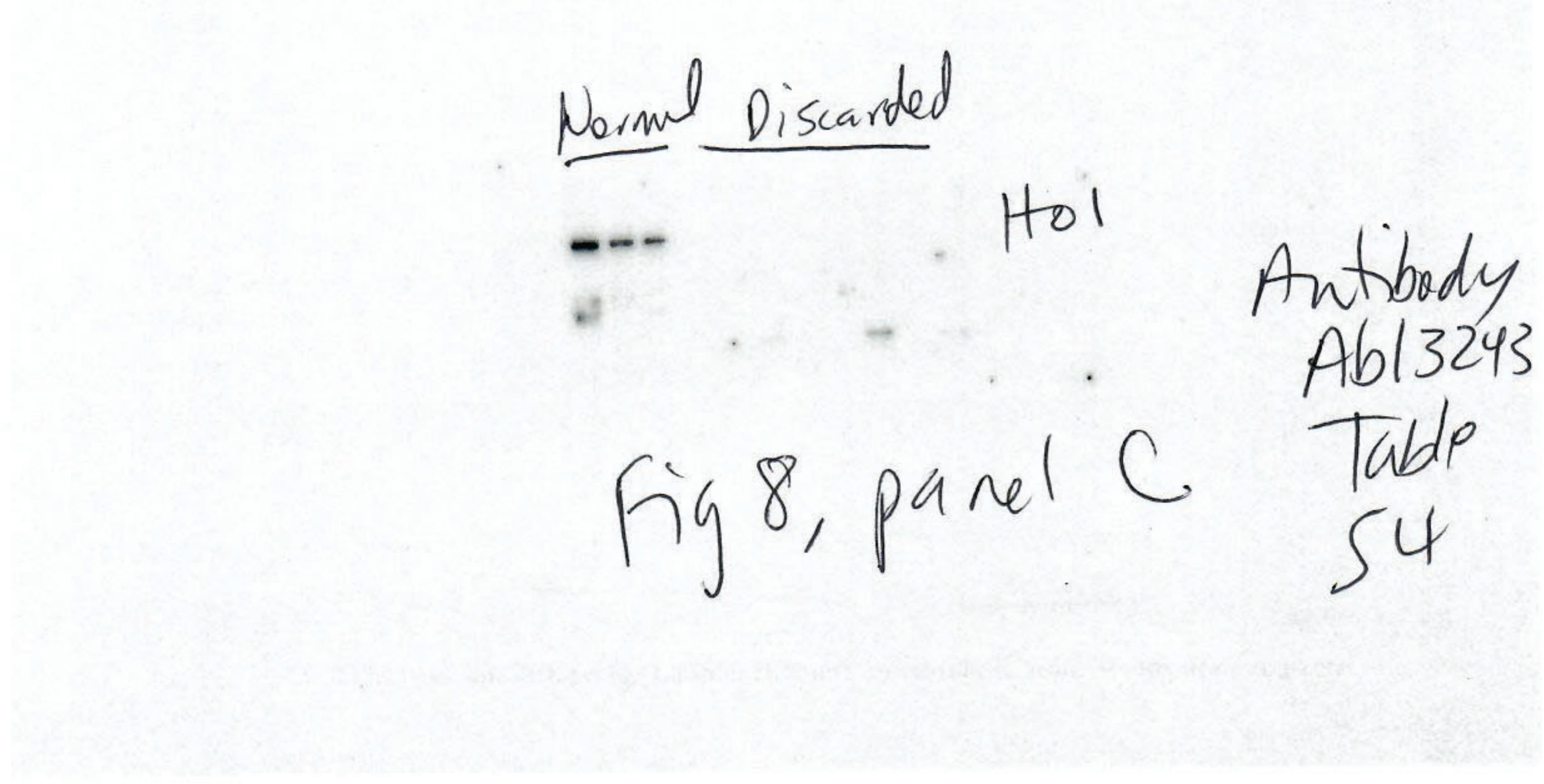

8.16.23 Full unedited gel Fig 1, HUR (Chemi) Ab, Replicate and Final

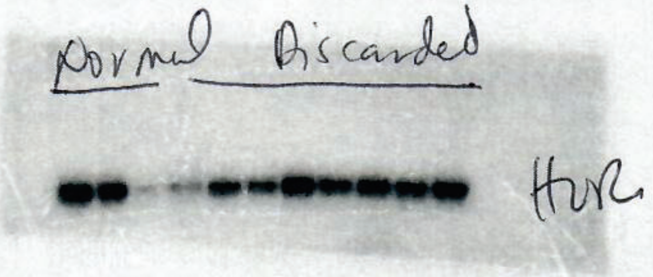

Antibody
SC-5261
Table S4

Fig 8, panel C

8.17.23 Full unedited gel Fig 1, rabbit VNC (Chemi) Ab, Replicate and Final

Normal Discarded

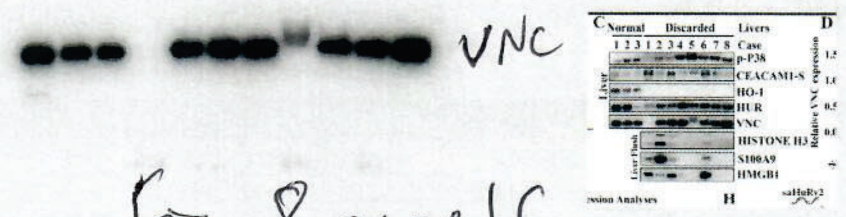

Fig 8, panel 1C

Antibody
E1E9V
Table S4

8.9.23 Full unedited gel Fig 1, Histone H3 Ab, Replicate and Final

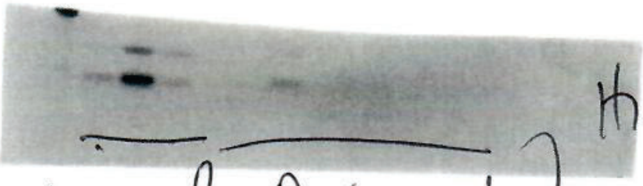

Fig. 8, panel C

Table S4
Antibody
44995

8.10.23 Full unedited gel Fig 1, S100A9 (Chemi) Ab, Replicate and Final

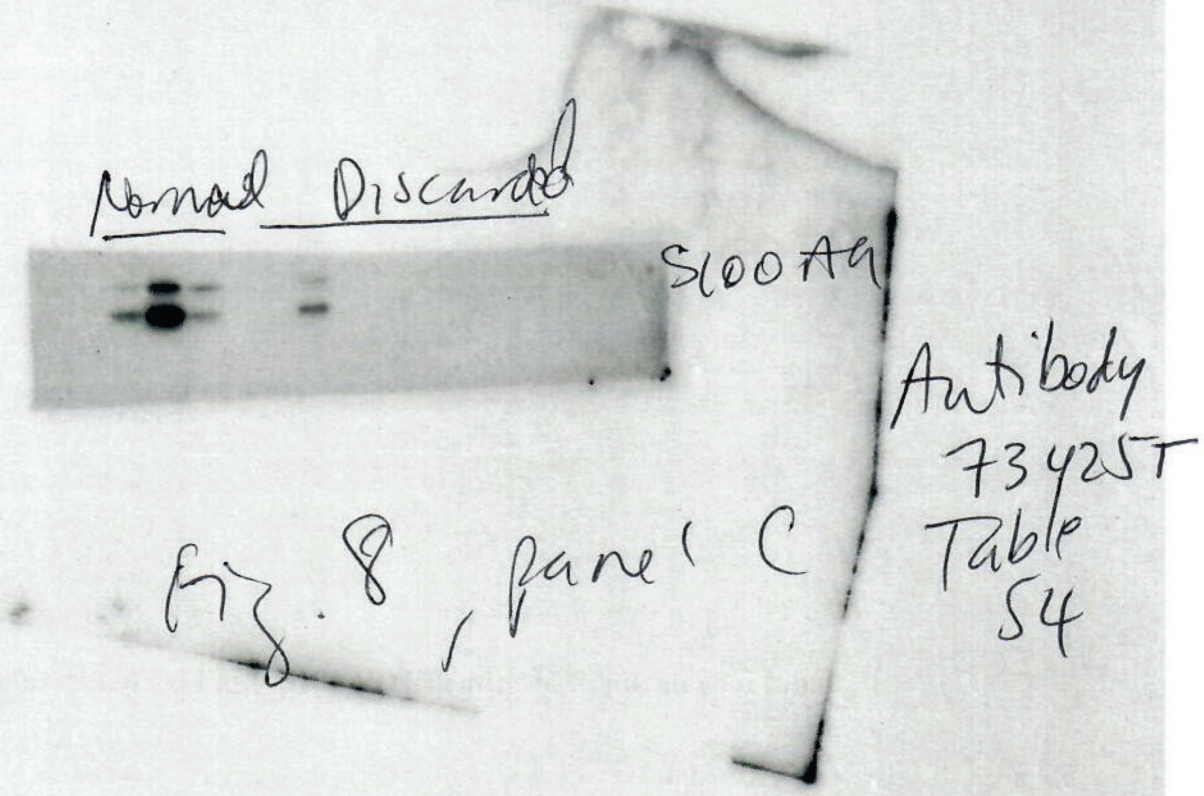

8.9.23 Full unedited gel Fig 1, HMGB1 (Chemi) Ab, Replicate and Final

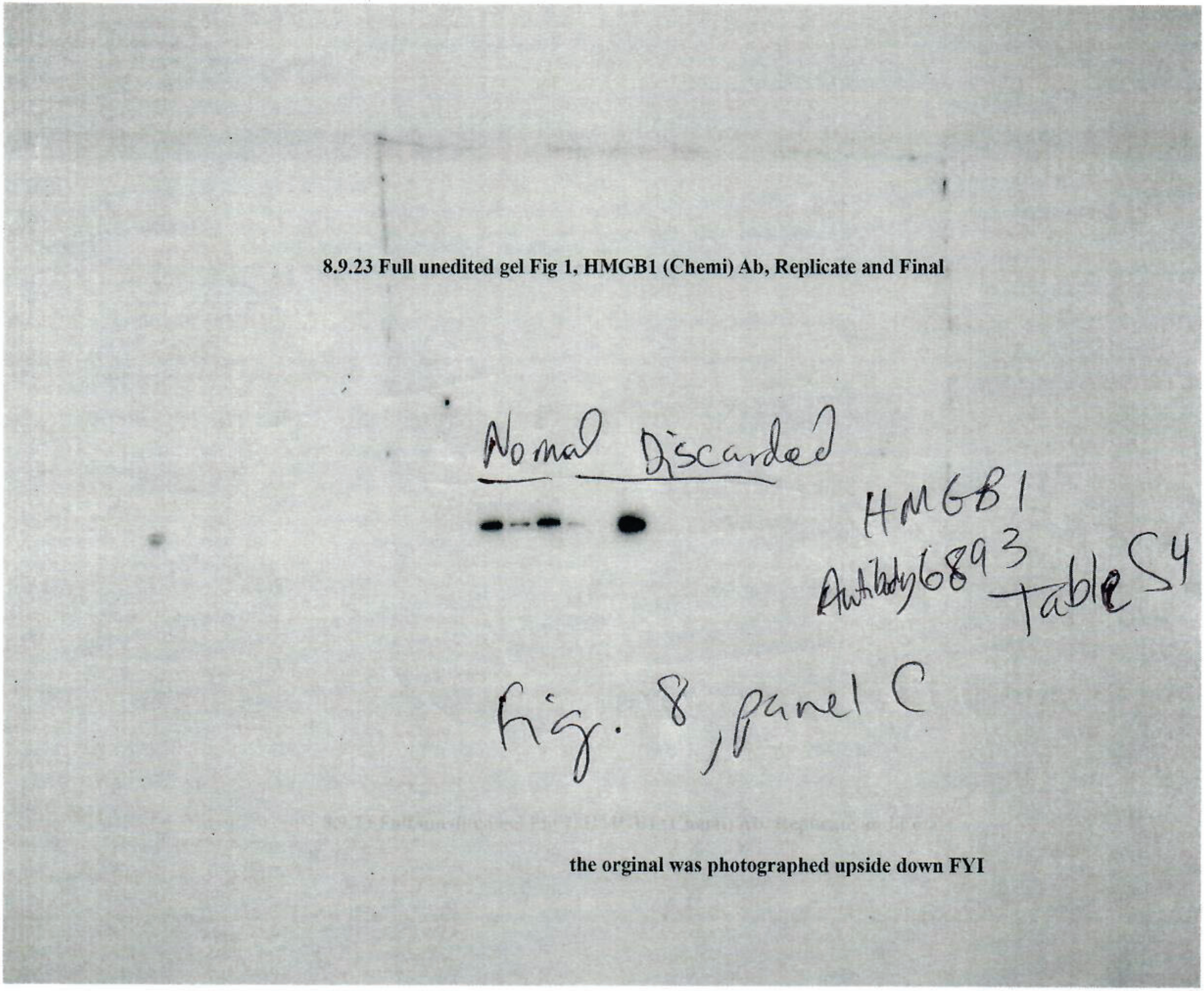

the orginal was photographed upside down FYI
